# Effects of resistance training on frailty-related outcomes in frail and pre-frail older adults: a systematic review and meta-analysis of randomized controlled trials

**DOI:** 10.3389/fpubh.2026.1896512

**Published:** 2026-10-22

**Authors:** Xiaodong Qian, Wei Huang, Lingzi Yao

**Affiliations:** 1 School of Physical Education, Hubei University of Education, Hubei, China; 2 Department of Gynecologic and Oncology, Hubei Cancer Hospital, Tongji Medical College, Huazhong University of Science and Technology, Hubei, China; 3 School of Physical Education, Guangzhou Maritime University, Guangdong, China

**Keywords:** frailty, handgrip strength, older adults, physical function, resistance training

## Abstract

**Background and Objective:**

Frailty is associated with muscle weakness, impaired physical function, and increased risk of disability and loss of independence, making preservation of muscle function an important public health goal for healthy aging. Resistance training may help address these deficits, but its effects in frail or pre-frail older adults remain uncertain. This systematic review and meta-analysis evaluated its effects on muscle strength, physical function, and frailty score.

**Methods:**

Seven electronic information sources were searched from inception to August 2026, with no language restrictions. Randomized controlled trials were included if they enrolled adults aged ≥60 years who were clearly diagnosed as frail or pre-frail using validated tools or comprehensive assessments. The intervention was limited to structured resistance training without combination with other exercise modalities. Random-effects models were used for meta-analysis, and the certainty of evidence was assessed using the GRADE approach.

**Results:**

Nine randomized controlled trials comprising 27 study arms and 566 participants were included. Low-certainty evidence suggested that resistance training may improve knee extensor strength (SMD = 1.30, 95% CI: 0.48 to 2.13). Very-low-certainty evidence showed possible improvements in handgrip strength (MD = 4.15 kg, 95% CI: 0.81 to 7.48) and gait speed (MD = 0.13 m/s, 95% CI 0.00 to 0.26), whereas the effects on Timed Up and Go (TUG), Short Physical Performance Battery (SPPB) and frailty score remained uncertain. For handgrip strength, gait speed, TUG, and SPPB, clinical importance remained uncertain because the 95% CI encompassed the corresponding reference thresholds for clinical importance. Between-study heterogeneity was substantial to considerable.

**Conclusion:**

Resistance training may improve knee extensor strength and may also benefit handgrip strength and gait speed in frail or pre-frail older adults, although the certainty of evidence is low to very low; effects on TUG, SPPB, and frailty score remain uncertain. Because muscle weakness and functional decline contribute to disability and loss of independence in later life, these findings may inform resistance-training strategies aimed at supporting muscle health and healthy aging. Further high-quality trials with standardized training prescriptions and longer-term follow-up are needed.

**Systematic review registration:**

https://www.crd.york.ac.uk/PROSPERO/view/CRD420261391816, identifier: CRD420261391816.

## Introduction

Frailty is a common clinical syndrome in geriatric medicine, characterized by a decline in multisystem physiological reserve and reduced tolerance to stressors (1). Unlike chronological aging alone, frailty represents an identifiable high-risk state and is closely associated with falls, hospitalization, admission to long-term care, mortality, reduced quality of life, and increased healthcare resource use (2–4). Frailty is not a fixed condition, as individuals may transition among robust, pre-frail, and frail states (5). Therefore, identifying and intervening in pre-frail or early frail older adults before substantial functional decline occurs is an important strategy for delaying functional deterioration and reducing adverse outcomes (6).

Exercise intervention is one of the most commonly recommended non-pharmacological strategies for frailty management (7). Current clinical practice guidelines recommend that frail older adults receive multicomponent physical activity interventions that include progressive resistance training; similar interventions may also be used in pre-frail older adults to delay or prevent the progression of frailty (8, 9). Resistance training is closely related to common problems in frail older adults, such as reduced muscle strength and impaired mobility, and may improve frailty-related outcomes by enhancing neuromuscular function, increasing muscle strength, and improving walking ability (10).

However, uncertainty remains regarding the clinical applicability of evidence on resistance training in frail or pre-frail older adults. Previous systematic reviews have often included older adults defined as frail or pre-frail using frailty assessment tools together with populations selected on the basis of sarcopenia, reduced muscle strength, or impaired physical performance (11, 12). This approach partly reflects the conceptual overlap between sarcopenia and physical frailty: weakness and slow gait speed are core diagnostic criteria in the widely used Fried physical frailty phenotype (13). However, sarcopenia mainly represents the muscle-related dimension of frailty and does not define frailty as a whole (14); moreover, frailty also includes constructs beyond physical frailty, such as cognitive frailty (15). Therefore, when populations defined by validated frailty tools are not distinguished from those selected only by subdimensions of frailty, the effects of resistance training in truly frail or pre-frail older adults remain difficult to interpret. It is therefore necessary to evaluate older adults with frailty or pre-frailty defined by validated frailty tools or comprehensive assessments separately, in order to improve the direct applicability of evidence to frailty management.

In addition, there is currently no universally accepted gold standard for diagnosing frailty (16). For intervention studies, it is particularly important to define the study population using validated comprehensive frailty assessment tools, rather than using sarcopenia, single physical performance indicators, disability, or comorbidity as surrogate criteria for frailty (17). Based on these considerations, this systematic review and meta-analysis included only randomized controlled trials that identified participants using validated tools or comprehensive assessment instruments explicitly used to define frail or pre-frail status. The aim was to evaluate the effects of structured resistance training on muscle strength, physical function and frailty score.

## Methods

### Study registration and reporting guidelines

This systematic review and meta-analysis was registered in PROSPERO, with the registration number CRD420261391816. The entire review process was conducted in accordance with the PRISMA 2020 reporting guidelines, including literature searching, study screening, data extraction, quality assessment, and result reporting (18).

### Literature search and study selection

PubMed, Embase, the Cochrane Central Register of Controlled Trials (CENTRAL), Web of Science, China National Knowledge Infrastructure (CNKI), ClinicalTrials.gov, and ProQuest were searched from database inception to August 2026. No language restrictions were applied. The revised search strategy was developed around frailty/pre-frailty, resistance training, and randomized controlled trials. Age-related terms were not used as a mandatory search block; instead, the age criterion (≥60 years) was applied during study screening. The intervention search was expanded to include commonly used free-text terms such as “resistance exercise,” “elastic band,” “TheraBand,” “progressive resistance,” and “strength exercise,” together with relevant database-specific subject headings. The complete search strategies for all sources are provided in Supplementary material S1. The search strategy was developed by the review team but was not formally peer reviewed by an information specialist or librarian using the PRESS guideline.

Study selection was independently performed by two reviewers. Records that were clearly irrelevant were first excluded based on titles and abstracts, after which full texts were assessed for eligibility. Any disagreement during screening was resolved through discussion between the two reviewers; if consensus could not be reached, a third reviewer made the final decision. To minimize the risk of missing potentially eligible studies, the reference lists of included studies and relevant systematic reviews were also manually searched. For reports for which full texts were not readily available, additional attempts were made to retrieve them through institutional library resources, bibliographic databases, Google Scholar, publisher websites, and other available online sources.

### Eligibility criteria

The eligibility criteria were defined according to the PICOS framework (19). Participants were older adults aged ≥60 years who were explicitly classified as frail or pre-frail. Frailty or pre-frailty had to be identified using validated assessment tools or comprehensive functional assessments, such as the Fried frailty phenotype, Frailty Index, Frailty Trait Scale-5, Clinical Frailty Scale, FRAIL scale, Edmonton Frailty Scale. The intervention was limited to structured resistance training without combination with other types of exercise intervention; no restrictions were placed on training mode, intensity, frequency, duration, or equipment type. Control conditions included health education, usual care, maintenance of daily activities, or low-intensity stretching only. Eligible studies were required to report at least one frailty- or physical function-related outcome, such as frailty score, Short Physical Performance Battery (SPPB), gait speed, Timed Up and Go (TUG), handgrip strength, knee extensor strength (KES). Only randomized controlled trials were included.

Studies were excluded if participants were not clearly classified as physically frail or pre-frail, or if the study population was defined solely by sarcopenia, cognitive impairment, social frailty, nutritional frailty, comorbidity, or disability without reporting physical frailty or pre-frailty. In addition, non-randomized studies, duplicate publications, studies without accessible full texts, and studies with data that could not be extracted or used to calculate effect sizes were not included in the quantitative synthesis.

### Data extraction

Two reviewers independently extracted data from each eligible randomized controlled trial. Extracted information included study characteristics, such as publication year, sample size, intervention duration, training frequency, training intensity, session duration, intervention details, and control condition; participant characteristics, such as sex, age, BMI, frailty diagnostic criteria, and baseline frailty score; and outcome data relevant to this review. When multiple publications were identified from the same RCT, they were treated as reports of a single trial. The report providing the most complete data for the eligible comparison was retained, whereas duplicate or secondary reports were excluded. For studies with missing or incomplete data, the corresponding authors were contacted up to three times within 3 weeks to obtain the relevant information.

### Measures of treatment effect

For continuous outcomes, treatment effects were calculated from change-from-baseline values between baseline and the end of the intervention. When studies reported mean changes and corresponding SDs, these data were extracted preferentially. When mean changes were not reported but baseline and postintervention means were available, change values were calculated as postintervention values minus baseline values. The treatment effect was defined as the change in the resistance training group minus the change in the control group. No endpoint-only values were used in the primary analyses. When the SD of the change score was unavailable, it was estimated as follows:


$$\begin{array}{l} \text{S}{\text{D}}_{\text{change}}\;=\;\sqrt{\text{S}{\text{D}}_{\text{baseline}}^{2}+\text{S}{\text{D}}_{\text{Post}}^{2}-2\times \text{r}\times \text{S}{\text{D}}_{\text{baseline}}\times \text{S}{\text{D}}_{\text{post}}} \end{array}$$

where *r* denotes the correlation between baseline and postintervention measurements. A correlation coefficient of 0.50 was assumed. Studies for which the SD could not be estimated from published data or obtained from the authors were excluded from the quantitative synthesis for the corresponding outcome.

Effect directions were standardized before effect sizes were calculated. For handgrip strength, knee extensor strength, gait speed and SPPB, higher values indicated better outcomes; therefore, positive effect estimates favored resistance training. For TUG completion time and frailty scores, lower values indicated better outcomes; therefore, negative effect estimates favored resistance training. In multi-arm trials, all eligible resistance-training arms were considered. For the primary overall meta-analysis, eligible intervention arms sharing a common control group were combined to avoid double-counting the control group.

### Risk of bias and certainty of the evidence assessment

Risk of bias was independently assessed by two reviewers using ROBUST-RCT, which evaluates six core items: random sequence generation, allocation concealment, blinding of participants, blinding of healthcare providers, blinding of outcome assessors, and outcome data not included in analysis. ROBUST-RCT was selected because it provides a concise and transparent assessment framework for randomized parallel-group trials. Item-level judgments were classified as definitely low, probably low, probably high, or definitely high risk. Because ROBUST-RCT does not prescribe an overall rating algorithm, we classified studies as overall low risk when all items were definitely/probably low and as overall high risk when ≥1 item was probably/definitely high (20). Two reviewers independently completed the assessments and recorded the rationale for each judgment, with disagreements resolved through discussion or adjudication by a third reviewer.

The certainty of evidence was assessed using the GRADE approach (21). Evidence from randomized controlled trials was initially rated as high certainty and was downgraded based on five domains: risk of bias, inconsistency, indirectness, imprecision, and publication bias. The certainty of evidence was finally classified as high, moderate, low, or very low.

### Statistical analysis

Meta-analyses were performed using the meta package in R. For continuous outcomes, sample size, mean, and standard deviation were extracted for the intervention and comparator groups, and effect estimates with 95% confidence intervals (CI) were calculated. Mean difference (MD) was used when outcomes were measured using the same scale or unit, whereas standardized mean difference (SMD) was used when different scales or units were used. SMD were expressed as Hedges' *g* with a small-sample correction and were interpreted as small (0.2), moderate (0.5), large (0.8), or very large (1.2) effects (22).

The primary analyses were conducted using random-effects models, with between-study variance (τ^2^) estimated using the restricted maximum likelihood (REML) method (23). The Hartung–Knapp method was applied to calculate confidence intervals and statistical tests for the pooled random-effects estimates. Heterogeneity was assessed using Cochran's Q, I^2^, and τ^2^. I^2^ values of 0%−40%, 30%−60%, 50%−90%, and 75%−100% were interpreted as potentially unimportant, moderate, substantial, and considerable heterogeneity, respectively (24). Ninety-five percent prediction intervals were also calculated for the primary meta-analyses to describe the range within which the true effect of a future comparable study might be expected to lie.

When change-score standard deviations were not reported, they were calculated from pre- and post-intervention standard deviations assuming a within-person correlation coefficient of *r* = 0.50. Sensitivity analyses were conducted using alternative correlation coefficients of *r* = 0.30 and *r* = 0.70 to assess the influence of this assumption. Robustness was further examined using leave-one-out analyses, in which each comparison was sequentially removed and the pooled random-effects estimate recalculated. In addition, an analysis excluding studies with structured active-exercise comparators was performed to assess whether differences in control-group activity exposure influenced the pooled estimates.

To explore potential sources of heterogeneity, we prespecified subgroup analyses by frailty status, training frequency, session duration, intervention duration, and training intensity. The planned training-frequency subgroup analysis was not undertaken because the data available for individual outcomes were too sparse and unevenly distributed across frequency categories to permit a meaningful between-subgroup comparison. This was a justified deviation from the registered protocol. Given the heterogeneous reporting of resistance-training intensity, the intensity analysis was exploratory and restricted to handgrip-strength comparisons in which intensity could be classified using sufficiently comparable %1RM-based prescriptions (>70% vs. ≤ 70% 1RM).

Random-effects models using REML estimation and Hartung–Knapp inference were retained within subgroup analyses, and differences between subgroups were assessed using the subgroup-difference test implemented in the meta package. Because fewer than 10 comparisons contributed to each outcome, funnel plots were used only for descriptive visual assessment of asymmetry. All statistical tests were two-sided, with *P* < 0.05 considered statistically significant.

## Results

### Study selection

A total of 2,889 records were identified through databases, registers, and other sources. After removing 594 duplicates, 2,295 records were screened by title and abstract, of which 2,260 were excluded. The full texts of 35 reports were sought for retrieval, and 4 could not be obtained. Of the 31 reports assessed for eligibility, 22 were excluded because of ineligible study design or randomization (*n* = 5), ineligible population (*n* = 6), ineligible intervention or comparator (*n* = 8), or duplicate/secondary publication (*n* = 3). Finally, 9 studies were included in the systematic review and meta-analysis. Detailed reasons for full-text exclusions are provided in Supplementary Table S2. The study selection process is shown in Figure 1.

**Figure 1:**
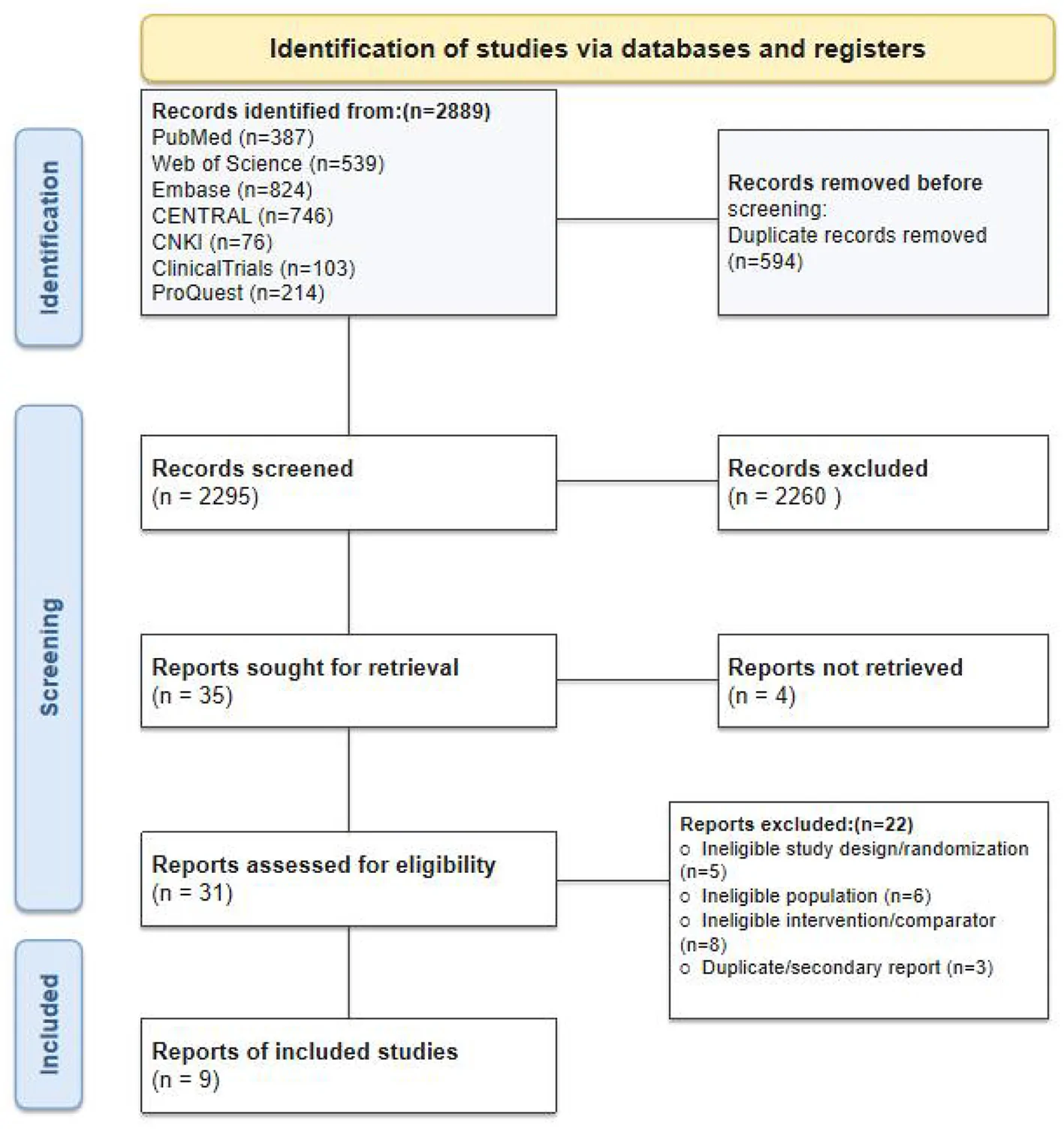
PRISMA flow diagram for literature search and selection.

### Study characteristics

The included studies comprised 27 study arms and 566 older participants, with a pooled mean age of 75.9 ± 10.6 years. Most studies diagnosed frailty or pre-frailty using the Fried/CHS frailty phenotype or modified versions of this criterion. In general, meeting 1–2 criteria was defined as pre-frailty, whereas meeting ≥3 criteria was defined as frailty. One study used the Tilburg Frailty Indicator to diagnose frailty syndrome in older adults with diabetes and also assessed frailty status using the FRAIL scale. The included studies were conducted in China, Portugal, Brazil, the United Kingdom, Turkey, and South Korea. Study settings included communities, hospitals, residential care homes, long-term care facilities, and social health support centers. All interventions were centered on resistance or strength training, including elastic band/TheraBand training, machine-based pneumatic resistance training, ankle-weight/dumbbell training, and chair-based exercise programs. Intervention duration ranged from 6 to 28 weeks, with a mean of approximately 14.9 weeks and a median of 12 weeks. Most interventions were delivered 2–3 times per week. Among studies reporting session duration, sessions lasted 25–60 min. Study characteristics are presented in Table 1, and intervention characteristics are presented in Supplementary Table S3.

**Table 1:** Characteristics of the included studies.

Study	Group	*n*	Frailty status	Frailty criteria	Baseline frailty	Age, y	BMI, kg/m^2^	Country/setting
Chen et al. (25)	RT	33	pre-frail	Fried phenotype; 1–2 = pre-frail	Fried score 1.30 ± 0.47	76.97 ± 5.19	22.79 ± 2.85	China; community
	UC	33	pre-frail	Fried phenotype; 1–2 = pre-frail	Fried score 1.42 ± 0.50	75.27 ± 5.98	22.64 ± 3.11	China; community
Coelho-Júnior and Uchida (26)	LSRT	11	pre-frail	Modified Fried phenotype	NR; Fried-defined pre-frail	65.0 ± 3.5	26.8 ± 5.7	Brazil; community
	HSRT	11	pre-frail	Modified Fried phenotype	NR; Fried-defined pre-frail	65.0 ± 2.8	24.5 ± 2.4	Brazil; community
	CG	10	pre-frail	Modified Fried phenotype	NR; Fried-defined pre-frail	65.0 ± 3.5	25.5 ± 3.3	Brazil; community
	LSRT	8	Frail	Modified Fried phenotype	NR; Fried-defined frail	75.0 ± 4.6	25.3 ± 3.1	Brazil; long-term care
	HSRT	11	Frail	Modified Fried phenotype	NR; Fried-defined frail	73.0 ± 7.5	24.6 ± 3.5	Brazil; long-term care
	CG	9	Frail	Modified Fried phenotype	NR; Fried-defined frail	75.0 ± 9.2	25.7 ± 2.4	Brazil; long-term care
Lai et al. (27)	RT	30	pre-frail	Fried criteria; 1–2 = pre-frail	NR; all pre-frail	65.3 ± 13.4	NR	China; hospital
	UC	30	pre-frail	Fried criteria; 1–2 = pre-frail	NR; all pre-frail	67.6 ± 11.9	NR	China; hospital
Lai et al. (28)	MV-LI RT	23	Frail	Fried phenotype; ≥3 = frail	NR; all frail	84.4 ± 5.0	23.9 ± 3.0	China; care agency
	MV-MI RT	23	Frail	Fried phenotype; ≥3 = frail	NR; all frail	87.5 ± 4.3	24.4 ± 4.1	China; care agency
	MV-HI RT	23	Frail	Fried phenotype; ≥3 = frail	NR; all frail	85.8 ± 8.7	23.8 ± 2.8	China; care agency
	HV-LI RT	23	Frail	Fried phenotype; ≥3 = frail	NR; all frail	85.0 ± 5.8	26.2 ± 9.2	China; care agency
	HV-MI RT	23	Frail	Fried phenotype; ≥3 = frail	NR; all frail	81.2 ± 7.7	24.0 ± 3.5	China; care agency
	HV-HI RT	23	Frail	Fried phenotype; ≥3 = frail	NR; all frail	85.4 ± 6.1	26.9 ± 12.8	China; care agency
	UC	23	Frail	Fried phenotype; ≥3 = frail	NR; all frail	81.7 ± 11.2	23.3 ± 4.3	China; care agency
Swales et al. (29)	RT	6	Frail	Fried phenotype; ≥3 = frail	3.33 ± 0.52	85.83 ± 7.83	25.22 ± 4.87	UK; residential care home
	UC	5	Frail	Fried phenotype; ≥3 = frail	3.20 ± 0.45	86.40 ± 7.20	27.83 ± 1.75	UK; residential care home
Sahin et al. (30)	RT	16	Frail	Physician-diagnosed Fried frailty	NR; all frail	84.18 ± 6.85	27.76 ± 5.95	Turkey; institution
	UC	16	Frail	Physician-diagnosed Fried frailty	NR; all frail	85.37 ± 4.70	27.43 ± 5.56	Turkey; institution
Ye et al. (31)	RT	48	Frail	TFI ≥ 4; FRAIL scale also used	TFI 8.15 ± 2.76	65.19 ± 5.02	23.94 ± 3.99	China; hospital
	UC	48	Frail	TFI ≥ 4; FRAIL scale also used	TFI 8.38 ± 2.57	66.88 ± 4.62	24.73 ± 3.52	China; hospital
Yoon et al. (32)	RT	20	pre-frail/frail	Cognitive frailty: CHS/Fried pre-frail or frail + CDR 0.5	Frailty score 1.63 ± 0.90	73.82 ± 4.37	24.86 ± 2.73	Korea; community
	UC	23	pre-frail/frail	Cognitive frailty: CHS/Fried pre-frail or frail + CDR 0.5	Frailty score 1.37 ± 0.56	74.03 ± 4.27	24.38 ± 3.30	Korea; community
Furtado et al. (33)	MCE	23	Frail	Fried PF index; ≥3 = frail	PFI 3.35 ± 1.42	79.92 ± 7.83	26.98 ± 3.97	Portugal; institution
	ESE	19	Frail	Fried PF index; ≥3 = frail	PFI 3.38 ± 1.72	81.15 ± 4.89	27.03 ± 8.67	Portugal; institution
	CG	18	Frail	Fried PF index; ≥3 = frail	PFI 3.37 ± 1.19	80.93 ± 10.10	27.45 ± 6.06	Portugal; institution

In Furtado et al. (33), only the ESE arm and the control group contributed to the meta-analysis; the MCE arm is shown for trial description only.

### Risk of bias

Among the 9 studies included in the risk-of-bias assessment, 4 were rated as having an overall low risk of bias (44.4%) and 5 as having an overall high risk of bias (55.6%) (Figure 2). High-risk judgments were most frequently observed for Outcome data not included in analysis (4 studies), followed by Allocation concealment (2 studies) and Blinding of outcome assessors (2 studies). No study was rated as high risk for Random sequence generation or Blinding of participants. One study was rated as high risk for Blinding of healthcare providers. These judgments reflected incomplete or inadequately reported concealment procedures, unblinded assessment of performance-based outcomes, post-randomization exclusions, attrition, or insufficient reporting of intention-to-treat analyses and missing-data handling. The distribution of risk-of-bias judgments across the six core ROBUST-RCT items is shown in Figures 2, 3, and study-specific judgments and rationales are provided in Supplementary material S5.

**Figure 2:**
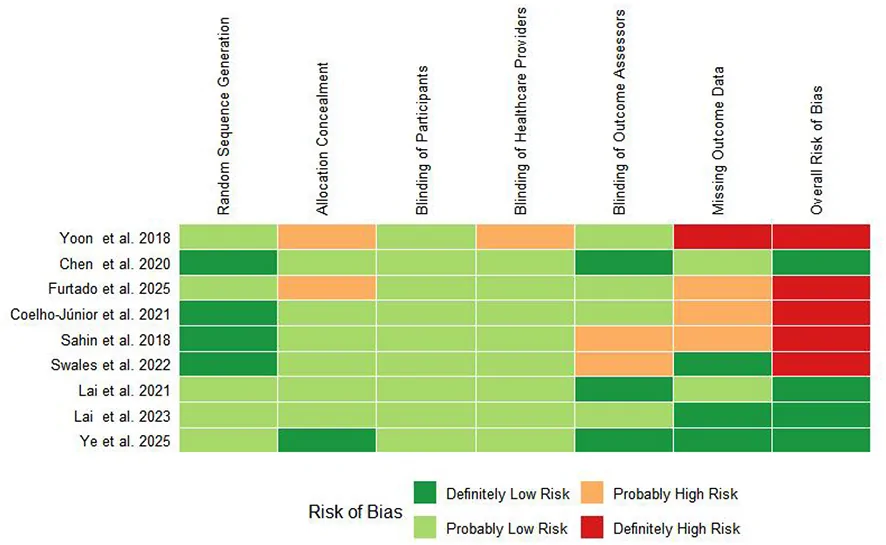
Risk of bias assessment of included studies.

**Figure 3:**
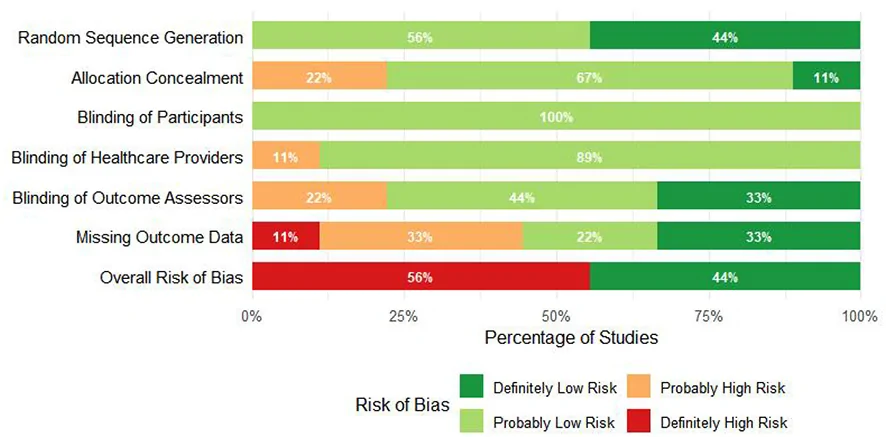
Distribution of risk of bias across domains.

### Certainty of evidence

The certainty of evidence ranged from low to very low across outcomes (Table 2). Evidence for knee extensor strength was rated as low certainty, whereas evidence for handgrip strength, gait speed, TUG, SPPB, and frailty score was rated as very low certainty. Downgrading was primarily due to risk of bias, inconsistency, and, for several outcomes, imprecision. No outcome was downgraded for indirectness. Publication bias was classified as undetected because fewer than 10 comparisons were available for each outcome, precluding reliable formal assessment. Detailed reasons for each downgrade are provided in the footnotes to Table 2.

**Table 2:** GRADE certainty of evidence for primary outcomes.

Outcome	No. of studies/ participants	Study design	Risk of bias	Inconsistency	Indirectness	Imprecision	Publication bias	Effect estimate	Absolute effect	Certainty of evidence
Grip strength	7 comparisons/353 participants	RCT	**Serious** ^**1**^	**Serious** ^**2**^	Not serious	**Serious** ^**3**^	Undetected^14^	MD = 4.15 kg, 95% CI: 0.81 to 7.48	4.15 kg higher (95% CI, 0.81 to 7.48 kg higher)	Very low certainty
Gait speed	6 comparisons/305 participants	RCT	**Serious** ^**1**^	**Serious4**	Not serious	**Serious** ^**5**^	Undetected^14^	MD = 0.13 m/s (95% CI, 0.00 to 0.26)	0.13 m/s higher (95% CI, 0.00 to 0.26 m/s higher)	Very low certainty
Timed Up and Go	4 comparisons/296 participants	RCT	**Serious** ^**1**^	**Serious** ^**6**^	Not serious	**Serious** ^**7**^	Undetected^14^	MD = −0.14 s (95% CI, −3.14 to 2.85)	0.14 s lower (95% CI, 3.14 s lower to 2.85 s higher)	Very low certainty
SPPB	3 comparisons/102 participants	RCT	**Very serious** ^**8**^	**Very serious** ^**9**^	Not serious	**Serious** ^**10**^	Undetected^14^	MD = 2.30 points (95% CI, −2.39 to 6.99)	2.30 points higher (95% CI, 2.39 points lower to 6.99 points higher)	Very low certainty
Knee extension strength	6 comparisons/340 participants	RCT	**Serious** ^**1**^	**Serious** ^**11**^	Not serious	Not serious	Undetected^14^	SMD = 1.30 (95% CI, 0.48 to 2.13)	1.30 SD higher (95% CI, 0.48 to 2.13 SD higher)	Low certainty
Frailty score	4 comparisons/190 participants	RCT	**Serious** ^**1**^	**Serious** ^**12**^	Not serious	**Serious** ^**13**^	Undetected^14^	SMD = −0.70 (95% CI, −1.76 to 0.35)	0.70 SD lower (95% CI, 1.76 SD lower to 0.35 SD higher)	Very low certainty

^1^ Downgraded one level for risk of bias because the contributing evidence included trials with methodological limitations, particularly concerns related to outcome data not included in analysis, allocation concealment, and/or blinding of outcome assessors; ^2^ Downgraded one level for inconsistency because considerable between-study heterogeneity was observed for handgrip strength (I^2^ = 85.6%); ^3^ Downgraded one level for imprecision because the 95% CI for handgrip strength (0.81 to 7.48 kg) crossed the 5.0-kg reference threshold for clinical importance; ^4^ Downgraded one level for inconsistency because substantial between-study heterogeneity was observed for gait speed (I^2^ = 78.0%); ^5^ Downgraded one level for imprecision because the 95% CI for gait speed (0.00 to 0.26 m/s) spanned the 0.10-m/s reference threshold for clinical importance; ^6^ Downgraded one level for inconsistency because substantial between-study heterogeneity was observed for TUG (I^2^ = 80.7%); ^7^ Downgraded one level for imprecision because the 95% CI for TUG (−3.14 to 2.85 s) was wide, crossed the null, and encompassed the 2.1-s reference threshold for clinical importance in both beneficial and unfavorable directions; ^8^ Downgraded two levels for risk of bias because the contributing SPPB evidence had major methodological limitations across multiple ROBUST-RCT items, including concerns regarding incomplete outcome data and assessor blinding; ^9^ Downgraded two levels for inconsistency because heterogeneity for SPPB was very high (I^2^ = 94.5%), indicating marked variation in effects across studies; ^10^ Downgraded one level for imprecision because the 95% CI for SPPB (−2.39 to 6.99 points) was very wide, crossed the null, and encompassed the 1.0-point reference threshold for clinical importance in both directions; ^11^ Downgraded one level for inconsistency because substantial between-study heterogeneity was observed for knee extensor strength (I^2^ = 77.2%); ^12^ Downgraded one level for inconsistency because substantial between-study heterogeneity was observed for frailty score (I^2^ = 60.5%); ^13^ Downgraded one level for imprecision because the 95% CI for frailty score (−1.76 to 0.35) was wide and crossed the null, leaving the magnitude and direction of the effect uncertain; ^14^ Publication bias was classified as undetected and was not downgraded because fewer than 10 comparisons were available for each outcome, precluding reliable formal assessment; no clear pattern of small-study effects was identified. When cumulative downgrading across GRADE domains exceeded three levels, the final certainty rating remained very low, as very low is the lowest certainty category in the GRADE framework.

### Muscle strength

Seven comparisons from six randomized controlled trials involving 353 participants reported handgrip strength. Very-low-certainty evidence suggested that resistance training may improve handgrip strength in frail or pre-frail older adults compared with control conditions (MD = 4.15 kg, 95% CI: 0.81 to 7.48; I^2^ = 85.6%; τ^2^ = 10.76). However, between-study heterogeneity was high, and the 95% prediction interval crossed the null (−4.59 to 12.88 kg) (Figure 4A).

**Figure 4:**
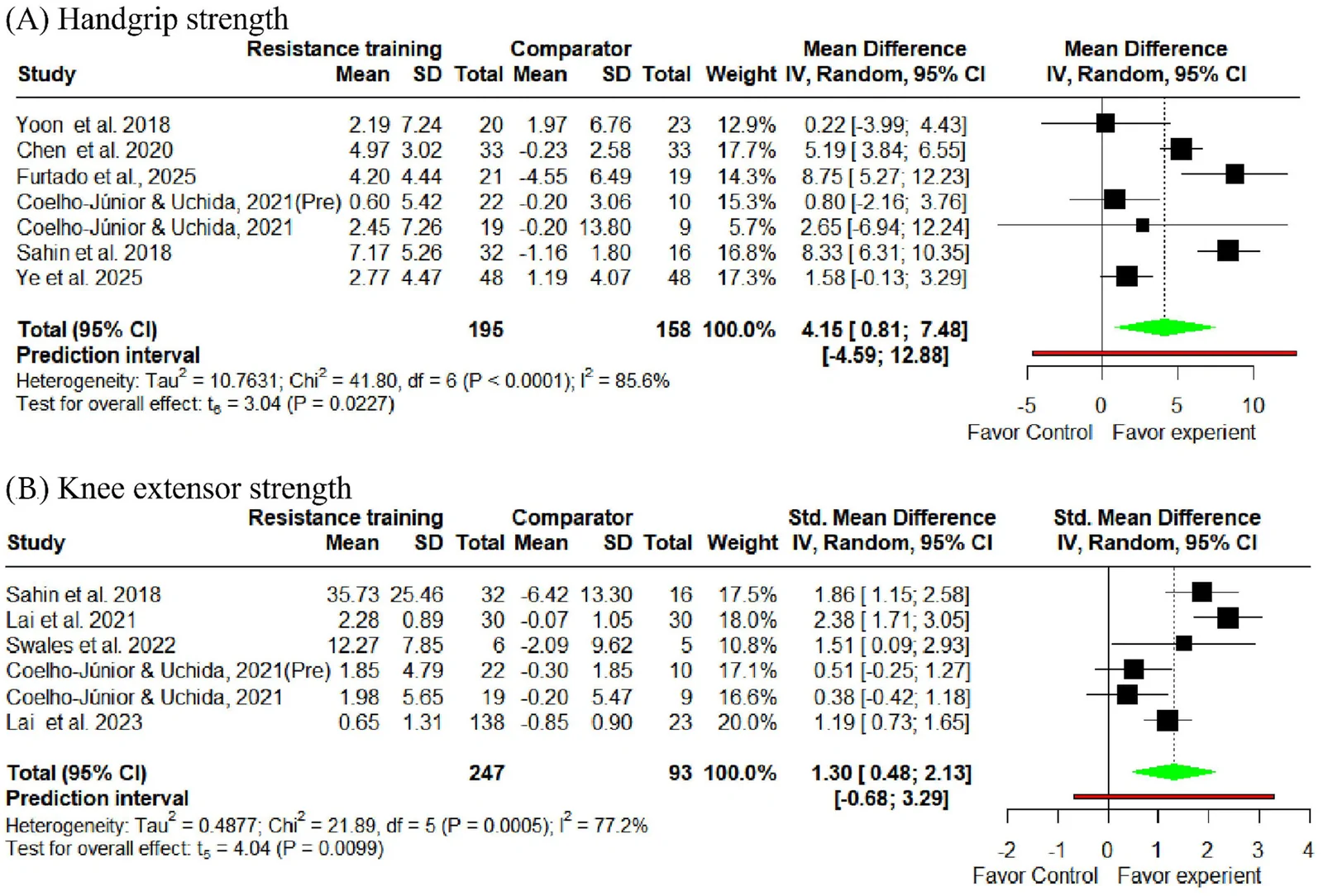
Forest plots of muscle strength: **(A)** handgrip strength; **(B)** knee extensor strength.

Six comparisons from five randomized controlled trials involving 340 participants reported knee extensor strength. Low-certainty evidence showed that resistance training may improve knee extensor strength in frail or pre-frail older adults (SMD = 1.30, 95% CI: 0.48 to 2.13; I^2^ = 77.2%; τ^2^ = 0.49). Between-study heterogeneity was high, and the 95% prediction interval also crossed the null (−0.68 to 3.29) (Figure 4B).

### Physical function

Six comparisons involving 305 participants reported gait speed. Very-low-certainty evidence suggested that resistance training may improve gait speed in frail or pre-frail older adults (MD = 0.13 m/s, 95% CI: 0.00 to 0.26; I^2^ = 78.0%; τ^2^ = 0.0067). However, between-study heterogeneity was high, the confidence interval ranged from a trivial effect to an effect exceeding the reference threshold for clinical importance, and the 95% prediction interval crossed the null (−0.11 to 0.36 m/s) (Figure 5A).

**Figure 5:**
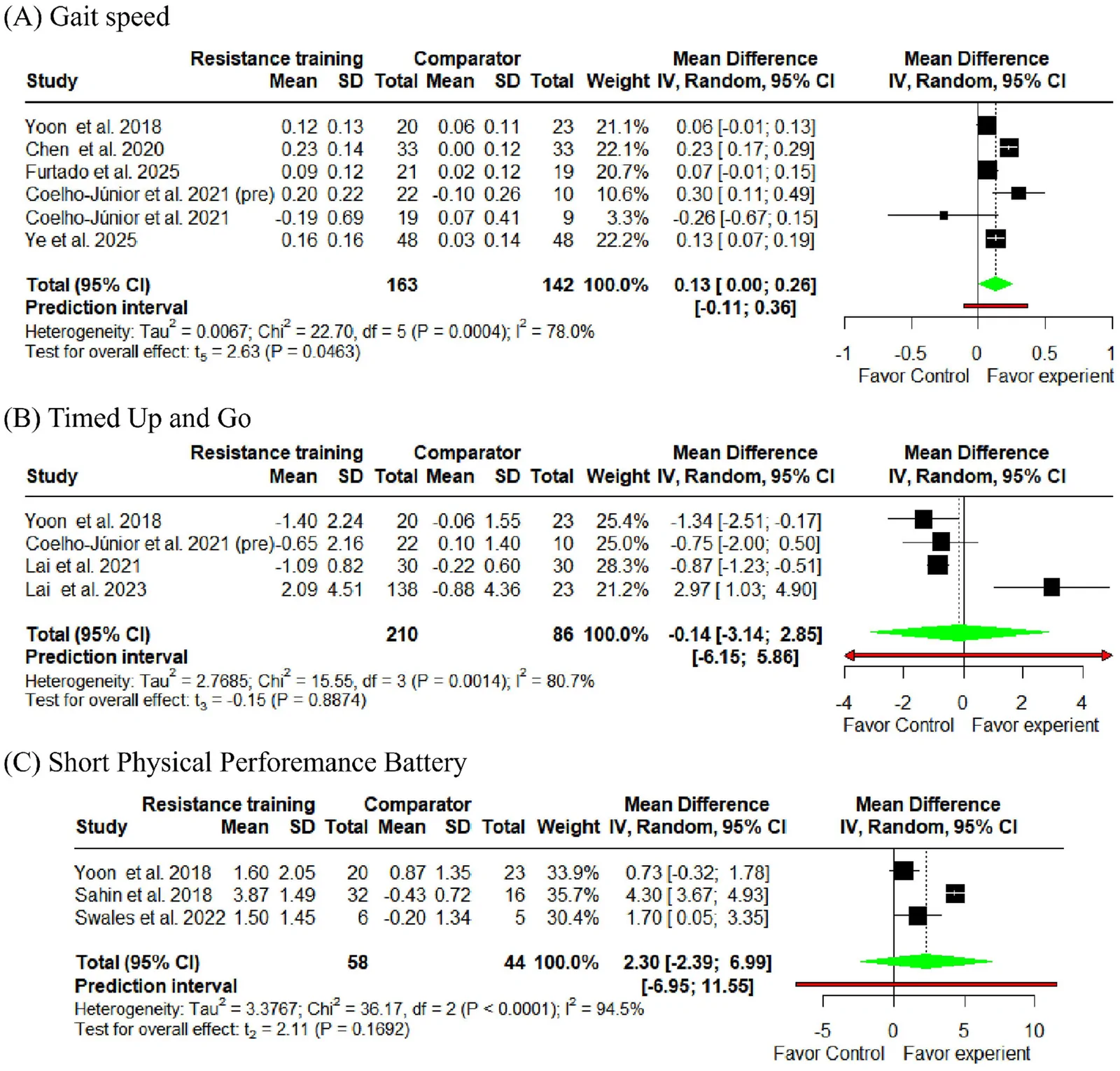
Forest plots of physical function: **(A)** Gait speed **(B)** Timed Up and Go **(C)** Short Physical Performance Battery.

Four comparisons involving 296 participants reported TUG. Very-low-certainty evidence showed that the effect of resistance training on TUG performance remains uncertain (MD = −0.14 s, 95% CI: −3.14 to 2.85; I^2^ = 80.7%; τ^2^ = 2.77). Between-study heterogeneity was high, and the 95% prediction interval was wide and crossed the null (-6.15 to 5.86 s) (Figure 5B).

Three comparisons involving 102 participants reported the SPPB. Very-low-certainty evidence showed that the effect of resistance training on SPPB score remains uncertain (MD = 2.30 points, 95% CI: −2.39 to 6.99; I^2^ = 94.5%; τ^2^ = 3.38). Between-study heterogeneity was very high, and the 95% prediction interval was wide and crossed the null (-6.95 to 11.55 points) (Figure 5C).

### Frailty score

Four comparisons involving 190 participants reported frailty score. Very-low-certainty evidence showed that the effect of resistance training on frailty score in frail or pre-frail older adults remains uncertain (SMD = −0.70, 95% CI: −1.76 to 0.35; I^2^ = 60.5%; τ^2^ = 0.14). Between-study heterogeneity was substantial, and the 95% prediction interval was wide and crossed the null (−2.17 to 0.76), indicating uncertainty in both the magnitude and consistency of the effect across comparable settings (Figure 6).

**Figure 6:**
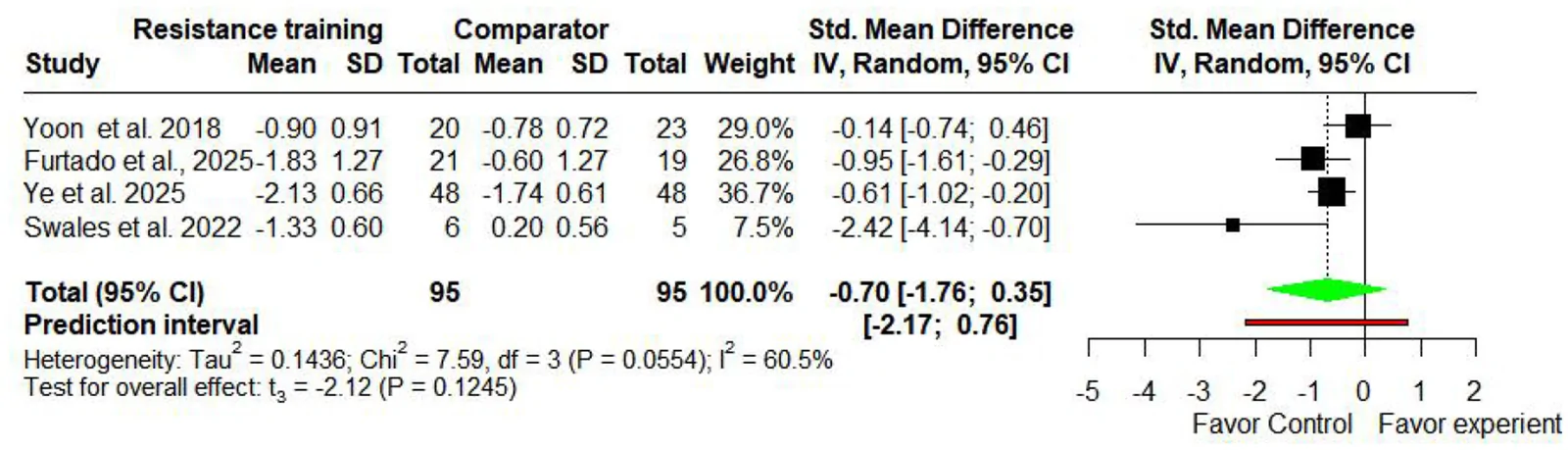
Forest plots of frailty score.

### Subgroup analyses

Subgroup analyses showed no evidence that the effects of resistance training on handgrip strength differed by intervention duration (>12 vs. ≤ 12 weeks; *P* for subgroup differences = 0.53), frailty status (*P* = 0.14), training intensity (>70% vs. ≤ 70% 1RM; *P* = 0.55), or session duration (>45 vs. ≤ 45 min; *P* = 0.26). Similarly, no statistically significant subgroup differences were identified for knee extensor strength according to intervention duration (*P* = 0.30) or frailty status (*P* = 0.88).

For gait speed, subgroup differences were not significant according to intervention duration (*P* = 0.34) or session duration (*P* = 0.70). However, the subgroup difference according to frailty status was statistically significant (*P* = 0.0036). The pooled effect on gait speed was positive in studies enrolling pre-frail participants (MD, 0.20 m/s; 95% CI, 0.01 to 0.39) and in studies enrolling combined frail and pre-frail populations (MD, 0.06 m/s; 95% CI, 0.00 to 0.13), whereas the estimate from the study enrolling frail participants alone was not statistically significant (MD, −0.26 m/s; 95% CI, −0.67 to 0.15). For Timed Up and Go, intervention duration did not significantly modify the pooled effect (*P* = 0.30). The prespecified subgroup analysis by training frequency was not performed because, within individual outcome datasets, comparisons were too sparse and unevenly distributed across frequency categories to support a meaningful between-subgroup comparison (Supplementary material S6).

### Sensitivity analyses and publication bias

Sensitivity analyses, including leave-one-out analyses, alternative assumed within-person correlations (r = 0.30 and 0.70) for imputed change-score standard deviations, and exclusion of studies with structured active-exercise comparators, generally supported the robustness of the findings, as the direction and magnitude of the pooled effects remained broadly consistent, although precision varied in some analyses (Supplementary material S7). Visual inspection of funnel plots did not suggest clear evidence of publication bias; however, this assessment should be interpreted cautiously because fewer than 10 studies were available for each outcome (Supplementary material S8).

### Adverse events, adherence, and attrition

Reporting of adverse events, adherence, and attrition across the 9 retained reports is summarized in Supplementary Table S4. Four reports stated that no adverse events or no intervention-attributable adverse effects occurred. Coelho-Júnior and Uchida (26) reported transient muscle fatigue during familiarization, joint pain in 2 participants, and epigastric discomfort or nausea in 1 participant during squat training; no falls occurred on training days. Ye et al. (31) reported adverse-event rates of 12.50% in the intervention group and 6.25% in the control group, with falls, sports injury, arrhythmia, elevated blood pressure, and dyspnoea recorded. Three reports did not present adverse-event data.

Numerical attendance or adherence data were reported in 3 studies. Attendance exceeded 95% in Coelho-Júnior and Uchida (26); the elastic-band strength-exercise arm in Furtado et al. (33) attended 72% of scheduled sessions; and Swales et al. (29) reported 82.4% attendance and 83.05% adherence to prescribed repetitions. Completion or attrition information was reported in 8 studies. Swales et al. (29) reported 100% retention, Lai et al. (28) reported no dropouts, and Coelho-Júnior and Uchida (26) reported 18 withdrawals among 78 randomized participants. Inconsistent participant accounting in several reports precluded a quantitative synthesis of adherence or attrition.

## Discussion

In this systematic review and meta-analysis of nine randomized controlled trials involving 566 older adults explicitly classified as frail or pre-frail, resistance training may improve knee extensor strength, although the certainty of this evidence was low. Possible benefits for handgrip strength and gait speed were supported by very-low-certainty evidence and were not consistently robust across sensitivity analyses. The effects on Timed Up and Go performance, Short Physical Performance Battery score, and frailty score remained uncertain. Substantial between-study heterogeneity and prediction intervals crossing the null for the pooled outcomes limited confidence in the consistency and clinical magnitude of the observed effects. Overall, the current evidence supports resistance training as a potential strategy for improving lower-limb strength in frail and pre-frail older adults, whereas its effects on broader physical function and frailty-related outcomes remain inconclusive.

Our findings refine previous evidence on resistance training in older adults with frailty. Although a network meta-analysis by Sun et al. (34) reported a relatively prominent effect of resistance training on frailty score among different physical activity interventions, the present review did not identify a clear effect on frailty score in participants explicitly classified as frail or pre-frail. Among the outcomes examined, the evidence was most supportive for knee extensor strength, for which resistance training was associated with improvement based on low-certainty evidence. Handgrip strength and gait speed also showed possible improvements, although the certainty of evidence for both outcomes was very low. In contrast, the effects on TUG, SPPB, and frailty score remained uncertain. Taken together, these findings suggest that the evidence for resistance training is currently clearer for muscle-strength outcomes than for broader measures of mobility, composite physical performance, or frailty severity.

One possible explanation for this outcome-specific pattern is the specificity of the training stimulus and outcome assessment. Many resistance-training programmes directly load the lower-limb extensor musculature, making knee extensor strength a relatively direct measure of training-related adaptation. By contrast, TUG and SPPB depend on the integration of multiple functional abilities beyond muscle strength, including balance, gait, chair-rise performance, turning, and task coordination (35, 36). Improvements in these outcomes may therefore require not only increased muscle strength but also balance, walking, and task-specific functional practice (37, 38). The present findings consequently support resistance training as an important component of frailty management, while suggesting that resistance training alone may not consistently improve multidimensional functional outcomes.

Differences from previous reviews should also be interpreted in relation to population definition and outcome characteristics. Nagata et al. (39) included older adults classified as frail or pre-frail using broader eligibility criteria, with frailty or pre-frailty identified not only by established frailty instruments but also by measures of physical performance, mobility, muscle strength, balance, endurance, fatigue, or physical activity. Importantly, Nagata et al. (39) reported a significant improvement in SPPB following resistance training, whereas the effect on SPPB remained uncertain in the present review. This contrast may partly reflect the broader definition of frailty used in their review, as well as differences in baseline functional status, intervention characteristics, and the composition of the included populations. These differences highlight the importance of clearly defining frailty when interpreting the effects of resistance training on specific physical-function outcomes.

Subgroup analyses of the examined training characteristics did not identify consistent effect modifiers and therefore did not support a single optimal resistance-training prescription. The subgroup difference in gait speed according to frailty status was exploratory. More favorable estimates were observed in studies enrolling pre-frail participants and mixed frail–pre-frail populations. However, because the frail-only subgroup was informed by a single comparison, this pattern should be regarded as hypothesis-generating rather than evidence of a differential effect according to frailty status. It is possible that mobility gains may be more readily achieved before frailty becomes advanced, when greater physiological reserve could facilitate the translation of strength gains into walking performance, but this interpretation requires confirmation in future studies. Evidence from individual trials further indicates that the effects of resistance training depend on the outcome targeted and on how the programme is delivered. Coelho-Júnior and Uchida (26) found that both low- and high-speed resistance training improved frailty-related outcomes, but low-speed training was more closely associated with strength gains and frailty reversal, whereas high-speed training showed greater benefits for mobility and dual-task performance. Sahin et al. (30) similarly reported improvements with both low- and high-intensity training, with a greater improvement in SPPB in the high-intensity group but limited between-group differences for other outcomes. Lai et al. (28) further suggested that higher training intensity or volume may enhance strength and six-minute walk performance, while lower-to-moderate doses may remain more feasible for some older adults.

Training intensity could be explored only to a limited extent. For handgrip strength, an exploratory subgroup analysis classified resistance-training programmes as >70% or ≤ 70% of one-repetition maximum and found no significant difference between intensity categories. However, this analysis was based only on the subset of trials in which training load could be expressed on a comparable %1RM scale. Across the broader evidence base, load prescription was reported inconsistently, with other studies using elastic-band color, perceived exertion, heart-rate-based targets, or providing insufficient information to permit standardized classification of resistance-training intensity (41). Therefore, the absence of a subgroup difference for handgrip strength should not be interpreted as evidence that training intensity is unimportant, nor can the available data establish an optimal intensity or an overall dose-response relationship between resistance-training intensity and frailty-related outcomes. Future trials should use clearly defined and progressive load prescriptions and consistently report training intensity and volume to facilitate more informative dose-response analyses.

## Clinical implications

From a clinical perspective, the present findings support resistance training as a potential component of frailty management, but they should not be interpreted as evidence that resistance training alone produces broad functional recovery. Reference thresholds for clinical importance were 0.10 m/s for gait speed and 1.0 point for SPPB (40), both derived from analyses including community-dwelling older adults, older adults with mobility limitations, and subacute stroke survivors; 2.1 s for TUG (41) was derived from patients undergoing surgery for lumbar degenerative disc disease; and 5.0 kg for handgrip strength (42) was derived from individuals with hemiparesis early after stroke. Because these reference thresholds were not established specifically in frail or pre-frail older adults, they were used only as reference values for interpreting clinical importance. Against these reference thresholds, the point estimate for gait speed (0.13 m/s) exceeded the 0.10-m/s reference threshold for clinical importance, but its 95% CI ranged from 0.00 to 0.26 m/s and the certainty of evidence was very low, leaving its clinical importance uncertain. The point estimate for handgrip strength (4.15 kg) was below the 5.0-kg reference threshold for clinical importance, although its 95% CI (0.81 to 7.48 kg) extended beyond it. The effect on TUG was small and highly uncertain (MD = −0.14 s; 95% CI, −3.14 to 2.85), while the SPPB point estimate exceeded the 1.0-point reference threshold for clinical importance but had a wide confidence interval that crossed the null. Thus, clinically important benefits for these outcomes cannot be established with confidence. Overall, resistance training may be most useful for targeting strength- and mobility-related limitations, although the certainty of evidence remains low to very low. For broader goals, such as improving composite physical function, quality of life, independence, or fall risk, resistance training may need to be combined with balance training, walking practice, task-specific exercise, nutritional support, or multicomponent geriatric care. Future trials should establish and report population-specific thresholds for clinical importance in frail and pre-frail older adults and include clinically interpretable outcomes such as frailty reversal, falls, activities of daily living, and maintenance of independence.

## Strengths and limitations

The main strength of this review is its strict eligibility criteria. Only randomized controlled trials were included, and participants had to be clearly identified as frail or pre-frail older adults using validated tools or comprehensive functional assessments. This reduced the risk of misclassification caused by using single indicators of muscle strength, muscle mass, disability, or comorbidity as substitutes for frailty. Second, this review evaluated muscle strength, mobility, comprehensive physical performance, and frailty score, providing a relatively broad overview of the effects of resistance training on frailty-related outcomes. Third, risk of bias assessment, GRADE evaluation, subgroup analyses, and multiple sensitivity analyses were conducted, helping to distinguish relatively stable findings from outcomes with greater uncertainty.

This review also has several limitations. First, the number of included trials and the total sample size were relatively limited, and some outcomes were informed by only a small number of studies, which may affect the precision of effect estimates and limit the interpretability of subgroup and sensitivity analyses. Second, intervention protocols varied across studies, including differences in training mode, frequency, intensity, duration, and progression, which may partly explain the heterogeneity observed for outcomes such as muscle strength, gait speed, and SPPB. Third, participants were recruited from different settings and clinical backgrounds, and their baseline functional status, frailty severity, and trainability may have differed, limiting the generalizability of the findings. Fourth, reporting of training prescriptions and safety or feasibility outcomes was insufficient, including details on progression, supervision, adverse events, dropout, training completion, and adherence; this limited conclusions about optimal training prescriptions and implementation safety. Fifth, several included trials had some risk of bias, mainly involving allocation concealment, blinding of outcome assessors, attrition, and missing data handling, which may have influenced the effect estimates.

## Conclusion

In frail or pre-frail older adults, resistance training may improve knee extensor strength, supported by low-certainty evidence, and may also benefit handgrip strength and gait speed, although the certainty of evidence for these outcomes is very low. The effects on TUG, SPPB, and frailty score remain uncertain. From a public health perspective, these findings may inform exercise strategies aimed at supporting muscle health and physical function in frail and pre-frail populations as part of broader efforts to promote healthy aging. Adequately powered trials in community and long-term care settings should evaluate progressive training protocols and long-term outcomes, alongside accessibility, adherence, and safety, to inform future implementation.

## Data Availability

The original contributions presented in the study are included in the article/Supplementary material, further inquiries can be directed to the corresponding author.
