# Supplementary material for: Effects of resistance training on frailty-related outcomes in frail and pre-frail older adults: a systematic review and meta-analysis of randomized controlled trials
Source: Front Public Health. 2026 Oct 22;14:1896512. doi: 10.3389/fpubh.2026.1896512 (PMC13735565; doi:10.3389/fpubh.2026.1896512)

Supplementary Appendix

Supplementary Table S1. Search strategies for electronic databases

The search strategies were adapted for PubMed, Embase, CENTRAL, and Web of Science using database-specific subject headings and free-text terms.

PubMed

Line	Search strategy
#1	("Resistance Training"[MeSH Terms] OR "resistance training"[Title/Abstract] OR "resistance exercise*"[Title/Abstract] OR "resistance exercise training"[Title/Abstract] OR "progressive resistance training"[Title/Abstract] OR "progressive resistance exercise*"[Title/Abstract] OR "strength training"[Title/Abstract] OR "strength exercise*"[Title/Abstract] OR "strengthening exercise*"[Title/Abstract] OR "muscle strengthening"[Title/Abstract] OR "muscle strengthening exercise*"[Title/Abstract] OR "weight training"[Title/Abstract] OR "weight lifting"[Title/Abstract] OR "weight-lifting"[Title/Abstract] OR "weight-bearing exercise*"[Title/Abstract] OR "elastic band*"[Title/Abstract] OR "elastic band exercise*"[Title/Abstract] OR "resistance band*"[Title/Abstract] OR "resistance band exercise*"[Title/Abstract] OR "elastic resistance"[Title/Abstract] OR "Theraband*"[Title/Abstract] OR "Thera-Band*"[Title/Abstract])
#2	("frailty"[MeSH Terms] OR "Frail Elderly"[MeSH Terms] OR frail*[Title/Abstract] OR prefrail[Title/Abstract] OR "pre-frail"[Title/Abstract] OR "pre-frailty"[Title/Abstract] OR "pre frail"[Title/Abstract] OR "pre frailty"[Title/Abstract])
#3	(randomized controlled trial[Publication Type] OR controlled clinical trial[Publication Type] OR randomized[Title/Abstract] OR randomised[Title/Abstract])
#4	#1 AND #2 AND #3

Embase

Line	Search strategy
#1	('resistance training'/exp OR 'resistance training':ti,ab,kw OR 'resistance exercise*':ti,ab,kw OR 'resistance exercise training':ti,ab,kw OR 'progressive resistance training':ti,ab,kw OR 'progressive resistance exercise*':ti,ab,kw OR 'strength training':ti,ab,kw OR 'strength exercise*':ti,ab,kw OR 'strengthening exercise*':ti,ab,kw OR 'muscle strengthening':ti,ab,kw OR 'muscle strengthening exercise*':ti,ab,kw OR 'weight training':ti,ab,kw OR 'weight lifting':ti,ab,kw OR 'weight-lifting':ti,ab,kw OR 'weight-bearing exercise*':ti,ab,kw OR 'elastic band*':ti,ab,kw OR 'elastic band exercise*':ti,ab,kw OR 'resistance band*':ti,ab,kw OR 'resistance band exercise*':ti,ab,kw OR 'elastic resistance':ti,ab,kw OR theraband*:ti,ab,kw OR thera-band*:ti,ab,kw)
#2	('frailty'/exp OR 'frail elderly'/exp OR frail*:ti,ab,kw OR prefrail*:ti,ab,kw OR pre-frail:ti,ab,kw OR pre-frailty:ti,ab,kw OR 'pre frail':ti,ab,kw OR 'pre frailty':ti,ab,kw)
#3	('randomized controlled trial'/exp OR 'controlled clinical trial'/exp OR random*:ti,ab,kw)
#4	#1 AND #2 AND #3

CENTRAL

Line	Search strategy
#1	MeSH descriptor: [Resistance Training] explode all trees
#2	"resistance training":ti,ab,kw OR "resistance exercise":ti,ab,kw OR "resistance exercise training":ti,ab,kw OR "progressive resistance training":ti,ab,kw OR "progressive resistance exercise":ti,ab,kw OR "strength training":ti,ab,kw OR "strength exercise":ti,ab,kw OR "strengthening exercise":ti,ab,kw OR "muscle strengthening":ti,ab,kw OR "muscle strengthening exercise":ti,ab,kw OR "weight training":ti,ab,kw OR "weight lifting":ti,ab,kw OR "weight-lifting":ti,ab,kw OR "weight-bearing exercise":ti,ab,kw OR "elastic band":ti,ab,kw OR "elastic band exercise":ti,ab,kw OR "resistance band":ti,ab,kw OR "resistance band exercise":ti,ab,kw OR "elastic resistance":ti,ab,kw OR Theraband:ti,ab,kw OR Thera-Band:ti,ab,kw
#3	("frail*":ti,ab,kw OR "prefrail":ti,ab,kw OR "pre-frail":ti,ab,kw OR "pre-frailty":ti,ab,kw OR "pre frail":ti,ab,kw OR "pre frailty":ti,ab,kw)
#4	#1 OR #2
#5	#3 AND #4

Web of Science

Line	Search strategy
#1	TS=("resistance training" OR "resistance exercise*" OR "resistance exercise training" OR "progressive resistance training" OR "progressive resistance exercise*" OR "strength training" OR "strength exercise*" OR "strengthening exercise*" OR "muscle strengthening" OR "muscle strengthening exercise*" OR "weight training" OR "weight lifting" OR "weight-lifting" OR "weight-bearing exercise*" OR "elastic band*" OR "elastic band exercise*" OR "resistance band*" OR "resistance band exercise*" OR "elastic resistance" OR "Theraband*" OR "Thera-Band*")
#2	TS=("frailty" OR "frail elderly" OR frail* OR prefrail OR "pre-frail" OR "pre-frailty" OR "pre frail" OR "pre frailty")
#3	TS=("randomized controlled trial" OR "randomised controlled trial" OR "controlled clinical trial" OR random*)
#4	#1 AND #2 AND #3

ClinicalTrials.gov

Line	Search strategy
#1	"resistance training" OR "resistance exercise*" OR "resistance exercise training" OR "progressive resistance training" OR "progressive resistance exercise*" OR "strength training" OR "strength exercise*" OR "strengthening exercise*" OR "muscle strengthening" OR "muscle strengthening exercise*" OR "weight training" OR "weight lifting" OR "weight-lifting" OR "weight-bearing exercise*" OR "elastic band*" OR "elastic band exercise*" OR "resistance band*" OR "resistance band exercise*" OR "elastic resistance" OR "Theraband*" OR "Thera-Band*"
#2	"frailty" OR "frail elderly" OR frail* OR prefrail OR "pre-frail" OR "pre-frailty" OR "pre frail" OR "pre frailty"
#3	#1 AND #2

ProQuest

Line	Search strategy
#1	"resistance training" OR "resistance exercise*" OR "resistance exercise training" OR "progressive resistance training" OR "progressive resistance exercise*" OR "strength training" OR "strength exercise*" OR "strengthening exercise*" OR "muscle strengthening" OR "muscle strengthening exercise*" OR "weight training" OR "weight lifting" OR "weight-lifting" OR "weight-bearing exercise*" OR "elastic band*" OR "elastic band exercise*" OR "resistance band*" OR "resistance band exercise*" OR "elastic resistance" OR "Theraband*" OR "Thera-Band*"
#2	"frailty" OR "frail elderly" OR frail* OR prefrail OR "pre-frail" OR "pre-frailty" OR "pre frail" OR "pre frailty"
#3	#1 AND #2

CNKI

Line	Search strategy
#1	抗阻训练 + 阻力训练 + 抗阻运动 + 力量训练 + 肌力训练 + 渐进性抗阻训练 + 渐进式抗阻训练 + 弹力带训练 + 弹力带运动 + 弹性阻力训练
#2	衰弱 + 衰弱前期 + 前衰弱 + 虚弱 + 体弱 + 衰弱综合征
#3	#1 AND #2

Abbreviations: CENTRAL, Cochrane Central Register of Controlled Trials; MeSH, Medical Subject Headings; TS, topic search.

Supplementary Table S2. Full-text reports excluded after eligibility assessment and reasons for exclusion

No.	Study report	Primary exclusion category	Specific reason for exclusion
1	The effect of resistance training for older adults with cognitive frailty: a randomized controlled trial	Ineligible comparator	Control group received structured active exercise.
2	Physical activity combined with resistance training reduces symptoms of frailty in older adults: A randomized controlled trial	Ineligible comparator	Control group received structured active exercise.
3	Research on the intervention effect of elastic-band resistant training program based on the COM-B model in the pre-frail elderly patients with type 2 diabetes mellitus	Ineligible comparator	Control group received structured active exercise.
4	The Effect of Elastic Band Flexible Resistance Training on Improving the Balance Ability of Pre-debilitated Elderly	Ineligible comparator	Control group received structured active exercise.
5	Effect of resistance training on older patients with chronic heart failure and frailty	Ineligible comparator	Control group received structured active exercise.
6	Effectiveness of the PROMUFRA program in pre-frail, community-dwelling older people: A randomized controlled trial	Ineligible intervention	Multicomponent exercise intervention rather than isolated resistance training.
7	The effect of functional circuit training on physical frailty in frail older adults: a randomized controlled trial	Ineligible intervention	Multicomponent exercise intervention rather than isolated resistance training.
8	Effect of low-load resistance training on physical function and depression state in elderly frail patients	Ineligible intervention	Intervention was not isolated resistance training.

No.	Study report	Primary exclusion category	Specific reason for exclusion
9	Effect of elastic band-based high-speed power training on cognitive function, physical performance and muscle strength in older women with mild cognitive impairment	Ineligible population	Participants were not explicitly classified as physically frail or pre-frail.
10	A randomized controlled trial of rehabilitation after hospitalization in frail older women: effects on strength, balance and mobility	Ineligible population	Participants were not explicitly classified as physically frail or pre-frail using an eligible frailty assessment.
11	Otago exercise programme for physical function and mental health among older adults with cognitive frailty during COVID-19: A randomised controlled trial	Ineligible population	Physical frailty or pre-frailty was not clearly established according to the prespecified eligibility criteria.
12	Resistance Exercise Intervention Restores Functional Capacity and Improves Frailty Biomarkers in Centenarians	Ineligible population	Participants were not explicitly classified as physically frail or pre-frail.
13	Effects of high-intensity progressive resistance training and targeted multidisciplinary treatment of frailty on mortality and nursing home admissions after hip fracture: a randomized controlled trial	Ineligible population	Participants were not explicitly classified as physically frail or pre-frail according to the prespecified criteria.
14	The effects of different types of physical exercise on physical and cognitive function in frail institutionalized older adults with mild to moderate cognitive impairment. A randomized controlled trial	Ineligible study design/randomization	Did not meet the prespecified eligible randomized controlled trial criteria.

No.	Study report	Primary exclusion category	Specific reason for exclusion
15	Handgrip strength does not represent an appropriate measure to evaluate changes in muscle strength during an exercise intervention program in frail older people	Ineligible study design/randomization	Not an eligible randomized controlled trial.
16	Impact of resistance exercise program on functional capacity and muscular strength of knee extensor in pre-frail community-dwelling older women: a randomized crossover trial	Ineligible study design/randomization	Randomized crossover design; not an eligible parallel-group RCT.
17	Impact of an exercise program on muscular and functional performance and plasma levels of interleukin 6 and soluble receptor tumor necrosis factor in prefrail community-dwelling older women: a randomized controlled trial	Ineligible study design/randomization	Did not meet the prespecified eligible randomized controlled trial criteria.
18	Effect of progressive resistance exercise on the frail elderly in nursing homes	Ineligible study design/randomization	Cluster allocation with only one cluster per treatment arm.
19	Effects of Resistance Band Exercises on Lower Limb Muscle Strength and Quality of Life in Pre-frail Elderly People	Duplicate/secondary report	Duplicate or secondary report of another trial cohort.
20	Study on the dose response relationship of resistance training on muscle strength and physical fitness of the frail elderly	Duplicate/secondary report	Duplicate or secondary report of another trial cohort.

Supplementary Table S3. Intervention characteristics of the included study groups

Study	Group	Intervention/comparator	Duration	Intensity/dose	Frequency	Session
Chen et al., 2020[29]	RT	Yellow elastic band; 8 exercises	8 wk	NA; 2×10-15	3×/wk	45-60 min
	UC	Usual activities; no structured exercise	8 wk	NA	NA	NA
Coelho-Junior & Uchida, 2021[30]	LSRT	Same 4 lower-limb exercises; slow concentric and eccentric phases (~2.5 s each)	16 wk	70-75% 1RM; 4×8-10	NR	NR

Study	Group	Intervention/comparator	Duration	Intensity/dose	Frequency	Session
	HSRT	Same 4 lower-limb exercises; concentric phase as fast as possible, eccentric ~2.5 s	16 wk	70-75% 1RM; 8×3-5	NR	NR
	CG	Flexibility training only	16 wk	NA	1×/wk	20 min
	LSRT	Same 4 lower-limb exercises; slow concentric and eccentric phases (~2.5 s each)	16 wk	70-75% 1RM; 4×8-10	NR	NR

Study	Group	Intervention/comparator	Duration	Intensity/dose	Frequency	Session
	HSRT	Same 4 lower-limb exercises; concentric phase as fast as possible, eccentric ~2.5 s	16 wk	70-75% 1RM; 8×3-5	NR	NR
	CG	Flexibility training only	16 wk	NA	1×/wk	20 min
Lai et al., 2021[31]	RT	5 lower-limb low-resistance exercises; later 0.5-1 kg ankle weights	12 wk	Borg RPE 11-13	3×/wk	30 min
	UC	Routine care and exercise education	12 wk	NA	NA	NA

Study	Group	Intervention/comparator	Duration	Intensity/dose	Frequency	Session
Lai et al., 2023[32]	MV-LI RT	Seated knee-extension Thera-Band exercise	12 wk	Yellow band 3.0 lb; 3×8	3×/wk	NR
	MV-MI RT	Seated knee-extension Thera-Band exercise	12 wk	Green band 4.6 lb; 3×8	3×/wk	NR
	MV-HI RT	Seated knee-extension Thera-Band exercise	12 wk	Blue band 5.8 lb; 3×8	3×/wk	NR
	HV-LI RT	Seated knee-extension Thera-Band exercise	12 wk	Yellow band 3.0 lb; 5×8	3×/wk	NR

Study	Group	Intervention/comparator	Duration	Intensity/dose	Frequency	Session
	HV-MI RT	Seated knee-extension Thera-Band exercise	12 wk	Green band 4.6 lb; 5×8	3×/wk	NR
	HV-HI RT	Seated knee-extension Thera-Band exercise	12 wk	Blue band 5.8 lb; 5×8	3×/wk	NR
	UC	Weekly exercise-related health education	12 wk	NA	1×/wk education	NR
Swales et al., 2022[37]	RT	Machine-based pneumatic RT; 7 exercises	6 wk	RPE 5–6 progressing to 7–8; 2×12	3×/wk	40 min
	UC	Wait-list control	6 wk	NA	NA	NA

Study	Group	Intervention/comparator	Duration	Intensity/dose	Frequency	Session
Sahin et al., 2018[33]	RT	Upper/lower-limb RT with ankle weights and dumbbells	8 wk	70% 1RM; 1×6-10	3×/wk	40 min
	UC	Usual activities; no exercise program	8 wk	NA	NA	NA
Ye et al., 2025[34]	RT	Seated Thera-Band PRT; upper limb, trunk and leg exercises	12 wk	50-70% HRR; 2-3×8-12	3×/wk	50 min
	UC	Diabetes care plus health education	12 wk	NA	NA	NA
Yoon et al., 2018[35]	RT	High-speed elastic-band RT; 8 exercises	16 wk	Blue band; RPE 12-13; 2-3×12-15	3×/wk	60 min

Study	Group	Intervention/comparator	Duration	Intensity/dose	Frequency	Session
	UC	Flexibility training only	16 wk	Not quantified	2×/wk	60 min
Furtado et al., 2025[36]	MCE	Chair-based multicomponent exercise; elastic-band endurance circuit (7-10 exercises)	28 wk	50-75% HRR; PES 1-4; 2-3×6-10	2-3×/wk	60 min
	ESE	Chair-based elastic-band muscle-strength exercise; 4-8 exercises	28 wk	TheraBand levels 1-3; PES 4-7; 2-3×12-15	2-3×/wk	60 min

Study	Group	Intervention/comparator	Duration	Intensity/dose	Frequency	Session
	CG	Usual lifestyle; no prescribed exercise	28 wk	NA	NA	NA

Supplementary Table S4. Reporting of adverse events, adherence, and attrition in retained study reports

Study report(s)	Adverse events / harms	Adherence / attendance	Attrition / completion	Reporting note
Chen et al. (2020)	Authors state that no adverse events occurred during the study.	Described as high; no aggregate attendance percentage. Attendance <85% was an exclusion criterion.	66 completed (33/group). Text reports 2 elastic-band withdrawals (personal reasons and/or low attendance) and 2 control losses (contact details changed).	The narrative later refers to one low-attendance elastic-band withdrawal; this is inconsistent with the earlier accounting.
Coelho-Junior & Uchida (2021)	Muscle fatigue during familiarisation; 2 participants reported joint pain and 1 reported epigastric discomfort/nausea during squat. No falls occurred on training days; falls on non-training days were recorded (HSRT 6; LSRT 4; control 4).	Attendance >95% in both pre-frail and frail samples.	78 randomised; 60 analysed (32 pre-frail; 28 frail). Eighteen withdrew. Reasons were detailed, including preference for another exercise group, personal reasons, 10RM testing, medication change, illness, stroke, urinary infection, and death.	The most complete harm reporting among the included reports; events are not all stated to be intervention-attributable.
Furtado et al. (2025)	No intervention-attributable adverse effects were reported, and no participant discontinued because of adverse effects.	Eligible elastic-band strength-exercise arm: 54/74 sessions (72%).	The report analysed 60 participants (multicomponent exercise 23, elastic-band strength exercise 19, control 18). It notes dropouts but does not provide an allocation-to-analysis flow or withdrawal reasons by the eligible elastic-band and control groups.	Furtado (2020) was removed and is not used to supplement safety, adherence, or attrition data in this table.
Lai et al. (2021)	NR. Safety monitoring and exercise-stopping criteria are described, but no adverse-event results are presented.	NR (nurse follow-up and video upload were used, but no attendance/compliance result was reported).	Text reports 1 exercise participant excluded for non-cooperation and 1 control participant who dropped out after myocardial infarction. Subsequent tables nevertheless retain n=30 per group.	Attrition and analysed denominators are internally inconsistent; retain this limitation in the risk-of-bias/feasibility narrative.
Lai et al. (2023)	NR. Participants were monitored and given stop criteria for symptoms, but no observed adverse-event data were reported.	No numerical rate. Poor compliance (<80% of scheduled sessions) was defined as dropout.	161 enrolled and randomised (23 in each of 7 groups); authors state that no participant dropped out during the intervention.	Completion is reported, but actual attendance and adverse-event counts are unavailable.
Swales et al. (2022)	No adverse events were reported during the feasibility trial.	Intervention (n=6): attendance 82.4% and adherence to prescribed	11 randomised (intervention 6; wait-list control 5); all completed baseline, post-intervention, and follow-up assessments.	Retention was 100%. The wait-list group's later exercise exposure had lower attendance/adherence

Study report(s)	Adverse events / harms	Adherence / attendance	Attrition / completion	Reporting note
		repetitions 83.05%. Excluding the ITT participant who did not train: 98.9% and 99.66%, respectively.	One intervention participant did not start training owing to unrelated health complications and medication changes but remained in the study for ITT.	because six scheduled sessions were cancelled by the facility (timetable clashes and norovirus procedures).
Sahin (Uk) et al. (2018)	Authors report no adverse effects during exercise.	NR.	66 randomised (22/group); 48 completed (16/group). Six per group did not receive/complete allocated intervention. Reasons shown in the flow diagram include severe heart/circulatory problems, fluid/electrolyte imbalance or hospitalisation, and discontinuation without a stated reason.	The statement of no adverse effects during exercise should not be equated with absence of all health events during the trial.
Ye et al. (2025)	Event counts were tabulated: intervention 12.50% versus control 6.25% (between-group P=0.294). Events listed were falls, sports injury, arrhythmia, elevated blood pressure, and dyspnoea.	NR.	96 analysed (48/group); no explicit participant-flow, withdrawal, or completion report was provided.	Safety table has internal arithmetic/reporting inconsistencies (e.g., displayed event counts/percentages and control total); report the authors' totals without recalculating them as participant-level risks.
Yoon et al. (2018)	NR.	Training was directly supervised by an exercise instructor to ensure adherence to the protocol; numerical attendance or adherence data were not reported.	The methods section reports 65 eligible participants allocated to high-speed resistance exercise (n=32) or control (n=33); 45 remained at 16 weeks (22 and 23, respectively). Reasons for loss to follow-up were not reported.	The Results section reports 66 randomised participants (33/33), whereas the baseline table presents 43 participants (20/23). The table uses the methods flow because it explicitly reports allocation and 16-week retention.

Notes: NR indicates not reported. “No adverse events” denotes an explicit author statement and is not inferred from nonreporting. Reported event counts may not represent unique participants or events attributable to training.

Supplementary Material S5. Risk-of-bias assessment of the retained studies

Note. Judgments are presented for each retained study across key risk-of-bias domains. The final column provides the overall study-level risk-of-bias judgment. Detailed justifications are provided in Supplementary Table S5.3.

Supplementary Table S5.1. Summary of domain-level risk-of-bias judgments

Domain	Definitely low risk	Probably low risk	Probably high risk	Definitely high risk	Low risk	High risk
Random sequence generation	4	5	0	0		
Allocation concealment	1	6	2	0		
Blinding of participants	0	9	0	0		
Blinding of healthcare providers	0	8	1	0		
Blinding of outcome assessors	3	4	2	0		
Missing outcome data	3	2	3	1		
Overall risk of bias					4	5

Supplementary Table S5.2. Risk-of-bias judgments by study

Study	Random sequence generation	Allocation concealment	Blinding of participants	Blinding of healthcare providers	Blinding of outcome assessors	Missing outcome data	Overall risk of bias
Yoon et al. 2018	Probably Low Risk	Probably High Risk	Probably Low Risk	Probably High Risk	Probably Low Risk	Definitely High Risk	High risk
Chen et al. 2020	Definitely Low Risk	Probably Low Risk	Probably Low Risk	Probably Low Risk	Definitely Low Risk	Probably Low Risk	Low risk
Furtado et al. 2025	Probably Low Risk	Probably High Risk	Probably Low Risk	Probably Low Risk	Probably Low Risk	Probably High Risk	High risk
Coelho-Júnior et al. 2021	Definitely Low Risk	Probably Low Risk	Probably Low Risk	Probably Low Risk	Probably Low Risk	Probably High Risk	High risk
Sahin et al. 2018	Definitely Low Risk	Probably Low Risk	Probably Low Risk	Probably Low Risk	Probably High Risk	Probably High Risk	High risk

Study	Random sequence generation	Allocation concealment	Blinding of participants	Blinding of healthcare providers	Blinding of outcome assessors	Missing outcome data	Overall risk of bias
Lai et al. 2021	Probably Low Risk	Probably Low Risk	Probably Low Risk	Probably Low Risk	Definitely Low Risk	Probably Low Risk	Low risk
Lai et al. 2023	Probably Low Risk	Probably Low Risk	Probably Low Risk	Probably Low Risk	Probably Low Risk	Definitely Low Risk	Low risk
Ye et al. 2025	Probably Low Risk	Definitely Low Risk	Probably Low Risk	Probably Low Risk	Definitely Low Risk	Definitely Low Risk	Low risk
Swales et al. 2022	Definitely Low Risk	Probably Low Risk	Probably Low Risk	Probably Low Risk	Probably High Risk	Definitely Low Risk	High risk

Supplementary Table S5.3. Justification for key judgments

Study	Justification for key judgments
Yoon et al. 2018	High risk was mainly driven by unclear allocation concealment and substantial, inconsistently reported missing outcome data. Although participants and providers were not blinded due to the nature of the exercise intervention, the assessed outcomes were objective/performance-based measures, so blinding concerns were judged less likely to cause major bias. However, no clear ITT analysis or missing-data handling was reported; therefore, the overall risk of bias was rated as high.
Chen et al. 2020	Low risk due to adequate random sequence generation using a random number table and acceptable allocation concealment through a sealed-box number draw. Although participants and providers were not blinded because of the exercise intervention, the assessed outcomes were mainly objective/performance-based measures, such as grip strength and walking speed. Outcome assessment was performed by a blinded assessor, and attrition was low and balanced between groups, with 66/70 participants completing the study.
Furtado et al. 2025	High risk was mainly driven by unclear allocation concealment and substantial missing outcome data. The study reported that 84 eligible participants were randomly allocated in a 1:1:1 ratio to MCE, ESE, and control groups, but the random sequence generation and concealment procedures were not clearly described. Participants and providers could not be blinded because the intervention involved chair-based multicomponent or elastic-band exercise, but the assessed objective outcomes, including salivary cortisol, salivary α-amylase, handgrip strength, and gait speed, were less vulnerable to expectation bias. Outcome measurement was partly protected because assessments were conducted by expert technicians and exercise instructors did not participate in data collection. However, only 60/84 randomized participants were analyzed after intervention, with group sizes reduced to MCE n=23, ESE n=19, and control n=18, indicating substantial and imbalanced attrition; therefore, overall risk was rated high.
Coelho-Júnior et al. 2021	High risk was mainly driven by substantial missing outcome data without clear ITT analysis. Randomization was adequate, using a computer-generated random number list by an independent researcher. Participants and providers were not blinded due to the resistance-training intervention, but outcomes were mainly objective/performance-based, including frailty criteria, physical performance, cognitive tests, and blood pressure. Outcome assessor blinding was incompletely reported, although blood pressure was blindly measured. However, 78 participants were randomized

Study	Justification for key judgments
	and only 60 were analyzed, with some exclusions potentially related to intervention or health status; therefore, overall risk was rated high.
Sahin et al. 2018	High risk was mainly driven by lack of assessor blinding and substantial post-randomization exclusions. Randomization was performed by lottery, and participants were not informed of the study hypothesis or assigned group. Participants and providers could not be fully blinded because of the exercise intervention, but the assessed objective/performance-based outcomes, such as muscle strength and SPPB, were less vulnerable to expectation bias. However, the same physiotherapist both assessed participants and supervised training, creating detection bias concerns. In addition, 66 participants were randomized but only 48 were analyzed, with 6 excluded from each group after allocation and no clear ITT analysis; therefore, overall risk was rated high.
Lai et al. 2021	Low risk was supported by random allocation using a random number table and separation of enrollment from assignment, although the paper also noted convenience sampling. Participants and providers could not be fully blinded due to the lower-limb resistance exercise intervention, but the assessed outcomes were objective/performance-based measures, including quadriceps strength, 6MWT, 30-s sit-to-stand, 8-ft up-and-go, and ActiGraph-measured energy expenditure. Outcome data collection was performed by investigators blinded to group allocation. Missing outcome data were minimal and balanced, with one participant excluded in each group; therefore, overall risk was rated low.
Lai et al. 2023	Low risk was supported by black-box randomization using sealed envelopes prepared by a third party, with balanced allocation across seven groups. Participants and providers could not be blinded because of the resistance-training intervention, but outcomes were objective/performance-based, including lower-limb muscle strength, 6MWT, 30-s sit-to-stand, and 8-foot up-and-go tests. Outcome assessor blinding was not clearly reported, but standardized objective measurements reduced detection bias concerns. No participants dropped out, all groups completed with 23 participants, and ITT analysis was reported; therefore, overall risk was rated low.
Ye et al. 2025	Low risk was supported by use of sealed, opaque, sequentially numbered envelopes for random allocation and explicit blinding of outcome assessors and statistical analysts. Participants and providers could not be fully blinded because of the Thera-Band resistance-training intervention, but the assessed outcomes were mainly objective/performance-based, including blood glucose, SPPB, handgrip strength, and usual gait speed. Both groups received consistent routine diabetes treatment, and no missing outcome data were reported, with analyses presented for 48 participants in each group; therefore, overall risk was rated low.
Swales et al. 2022	High risk was mainly driven by unblinded outcome assessment. The trial used 1:1 permuted block randomization with a computer-generated random number generator, and allocation was not revealed until consent, eligibility screening, and baseline measures had been completed. Participants, care staff, and the well-being team were aware of allocation because of the exercise intervention, but the assessed outcomes were mainly objective/performance-based measures and standardized testing protocols were used. However, the same researcher delivered the intervention and completed all post-intervention and follow-up assessments without blinding, which could influence performance-based outcomes such as strength and functional capacity. Missing outcome data were not a concern because all 11 randomized participants completed outcome assessments and were included in intention-to-treat analyses.

Abbreviations: ITT, intention-to-treat; RCT, randomized controlled trial; SPPB, Short Physical Performance Battery; 6MWT, 6-min walk test.

Supplementary Material S6. Subgroup analyses

Figures are organized by outcome domain. Muscle strength outcomes include handgrip strength and knee extensor strength (KES).

Figure S6.1. Subgroup analysis of handgrip strength according to intervention duration.

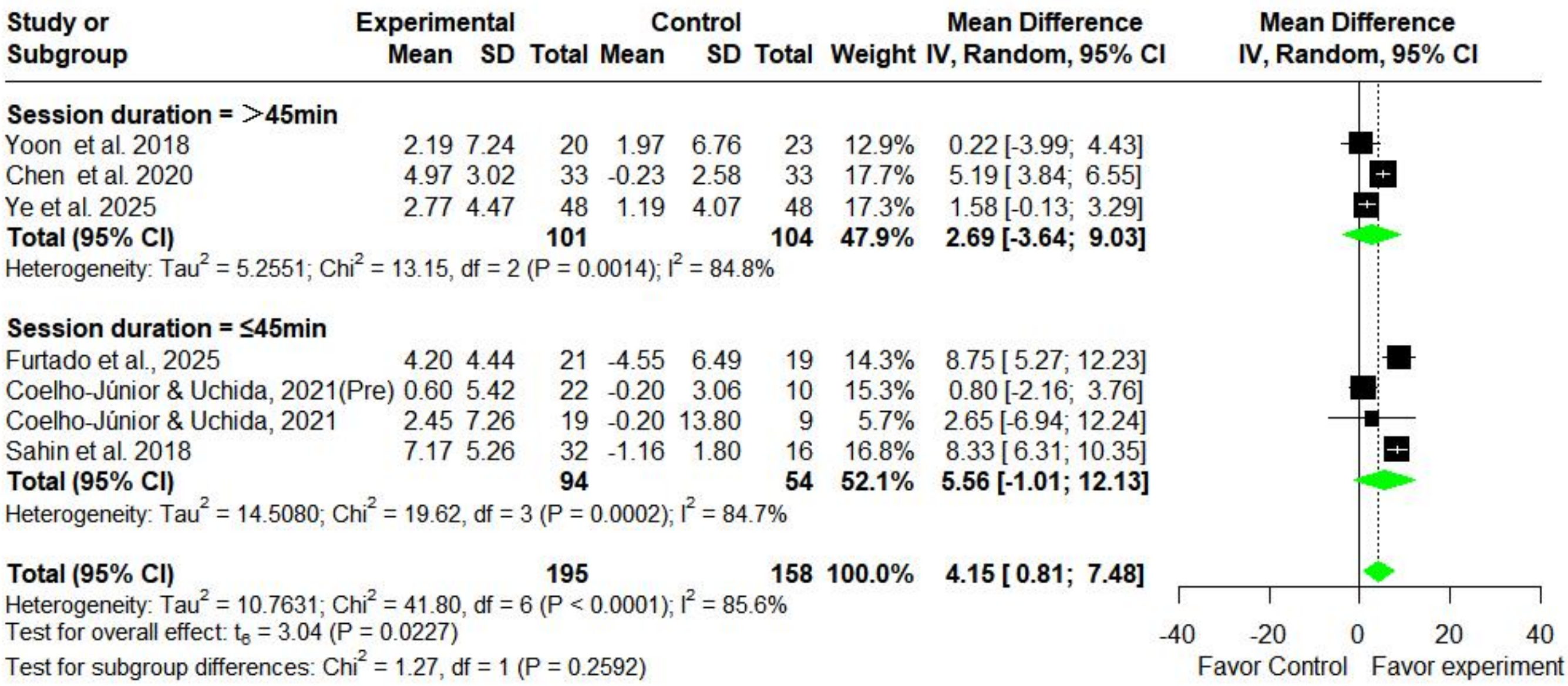

Figure S6.2. Subgroup analysis of handgrip strength according to frailty status.

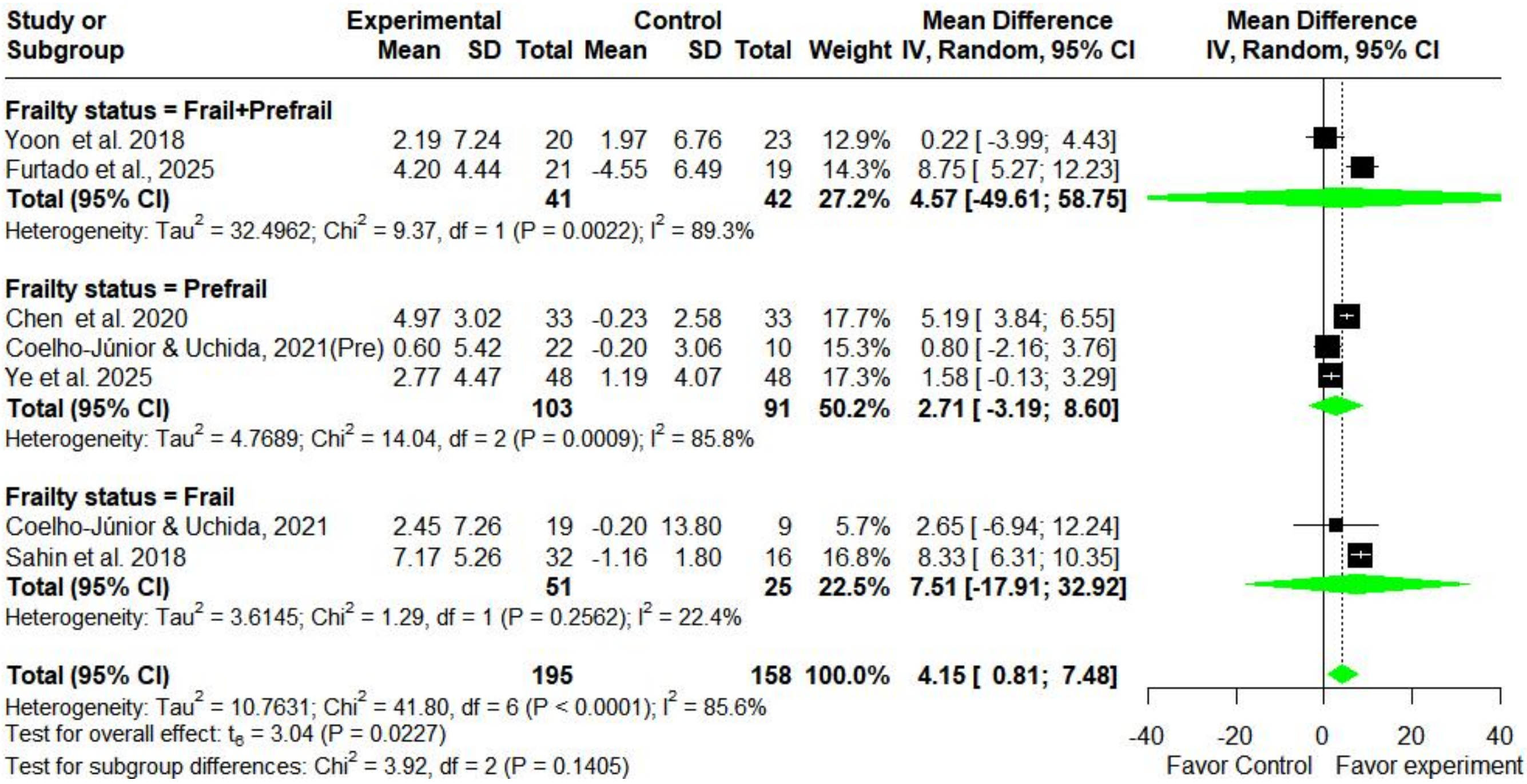

Figure S6.3. Subgroup analysis of handgrip strength according to intervention intensity.

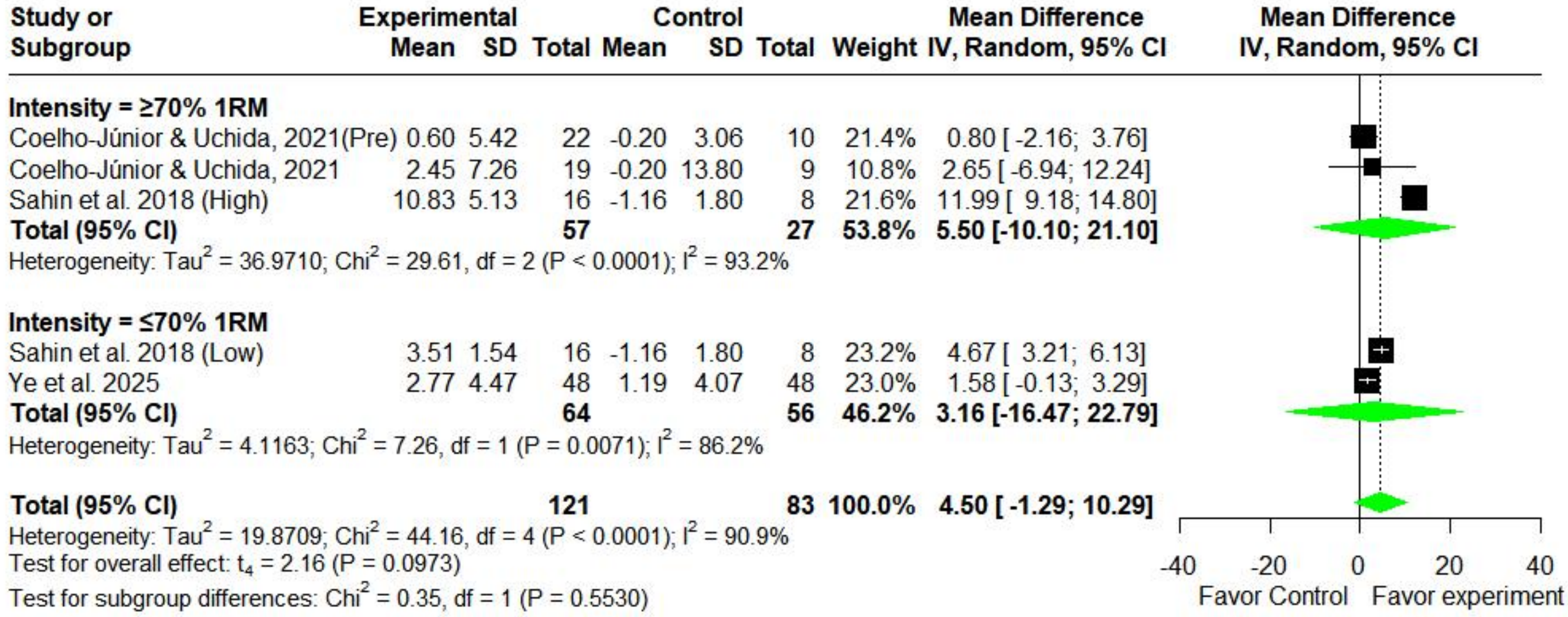

Figure S6.4. Subgroup analysis of handgrip strength according to session duration.

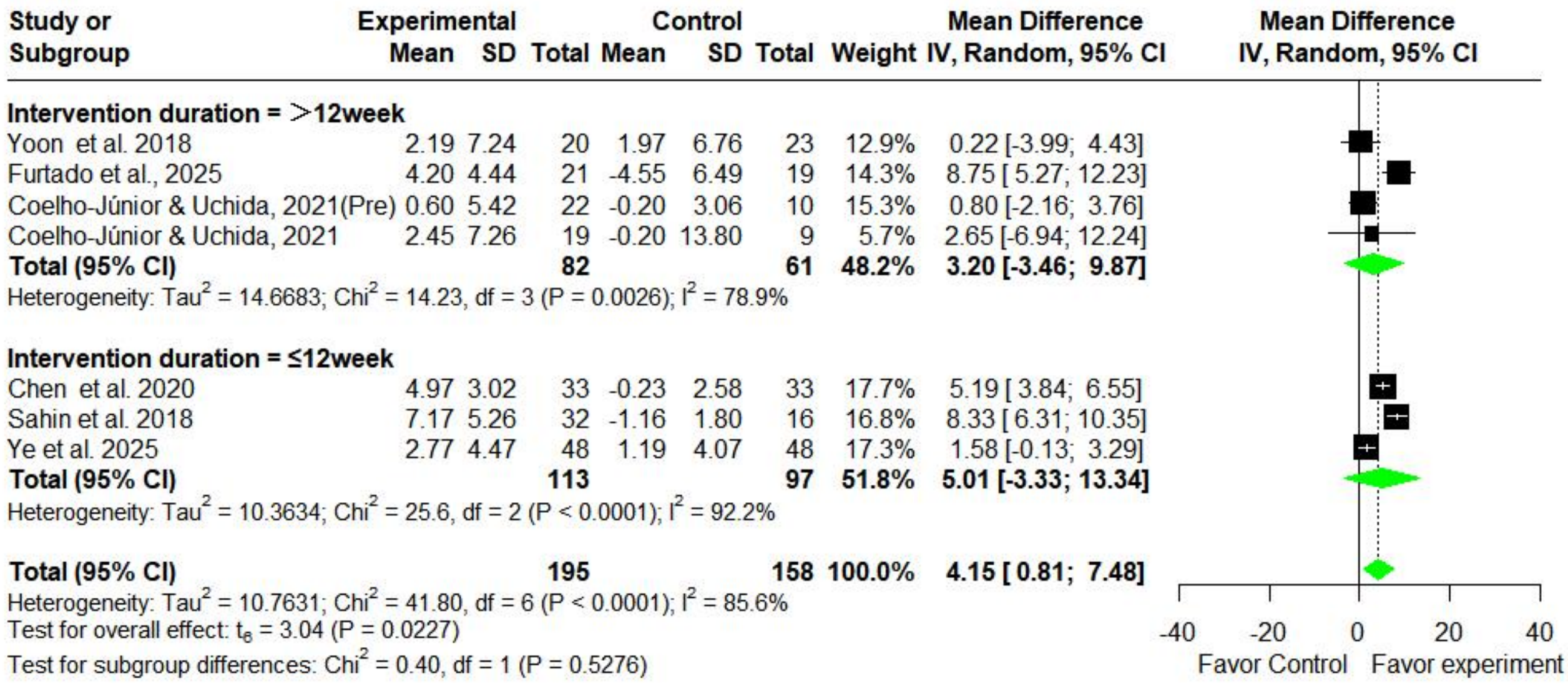

Figure S6.4. Subgroup analysis of knee extensor strength according to session duration.

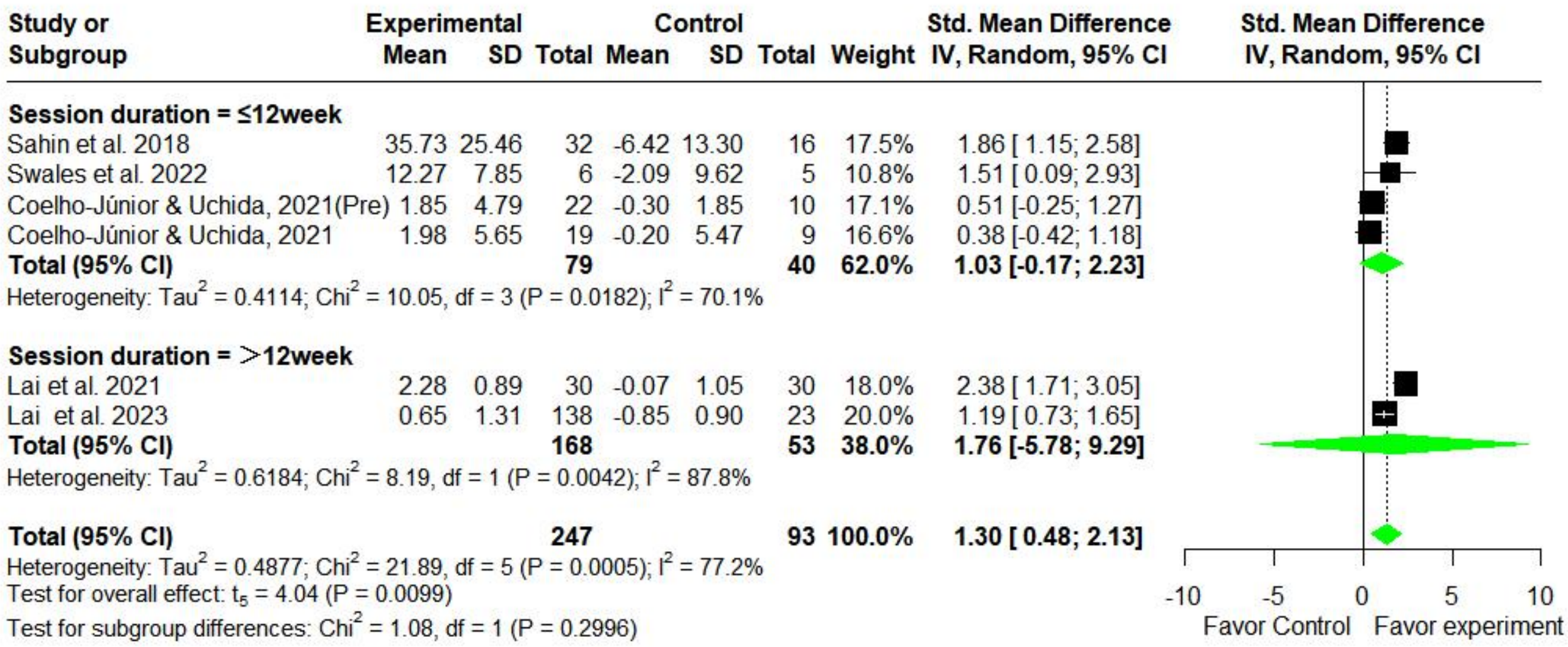

Figure S6.6. Subgroup analysis of knee extensor strength according to frailty status.

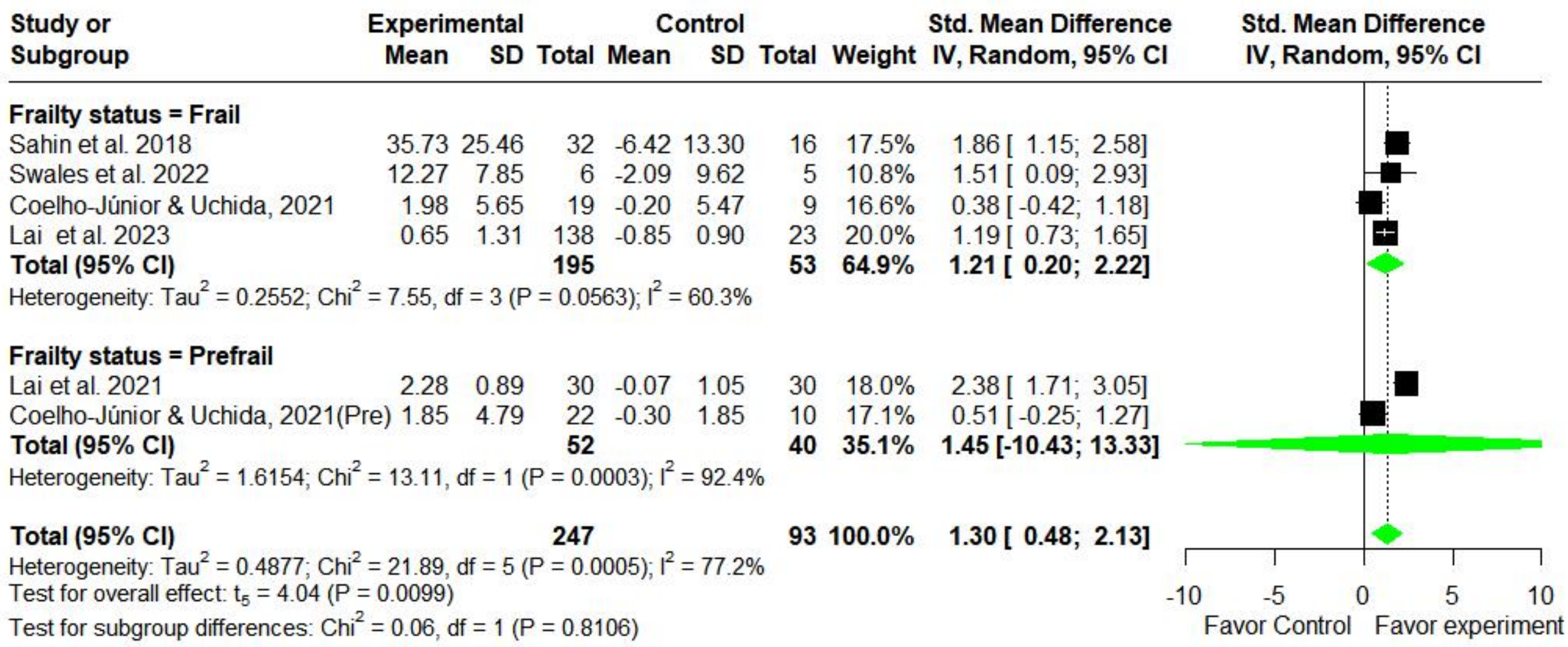

Supplementary Material S6.2. Subgroup analyses for physical function

Physical function outcomes were organized by gait speed and TUG.

Figure S6.7. Subgroup analysis of gait speed by intervention duration.

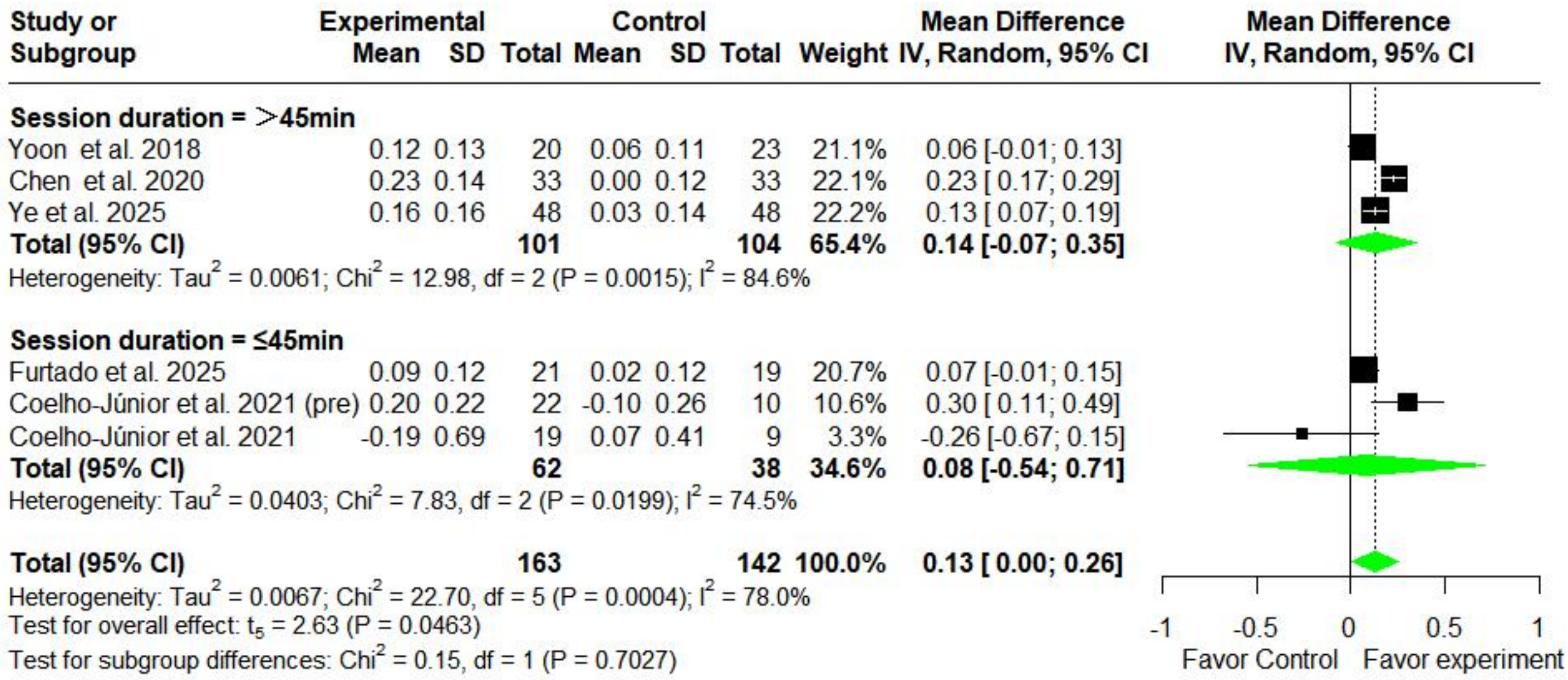

Figure S6.8. Subgroup analysis of gait speed by frailty status.

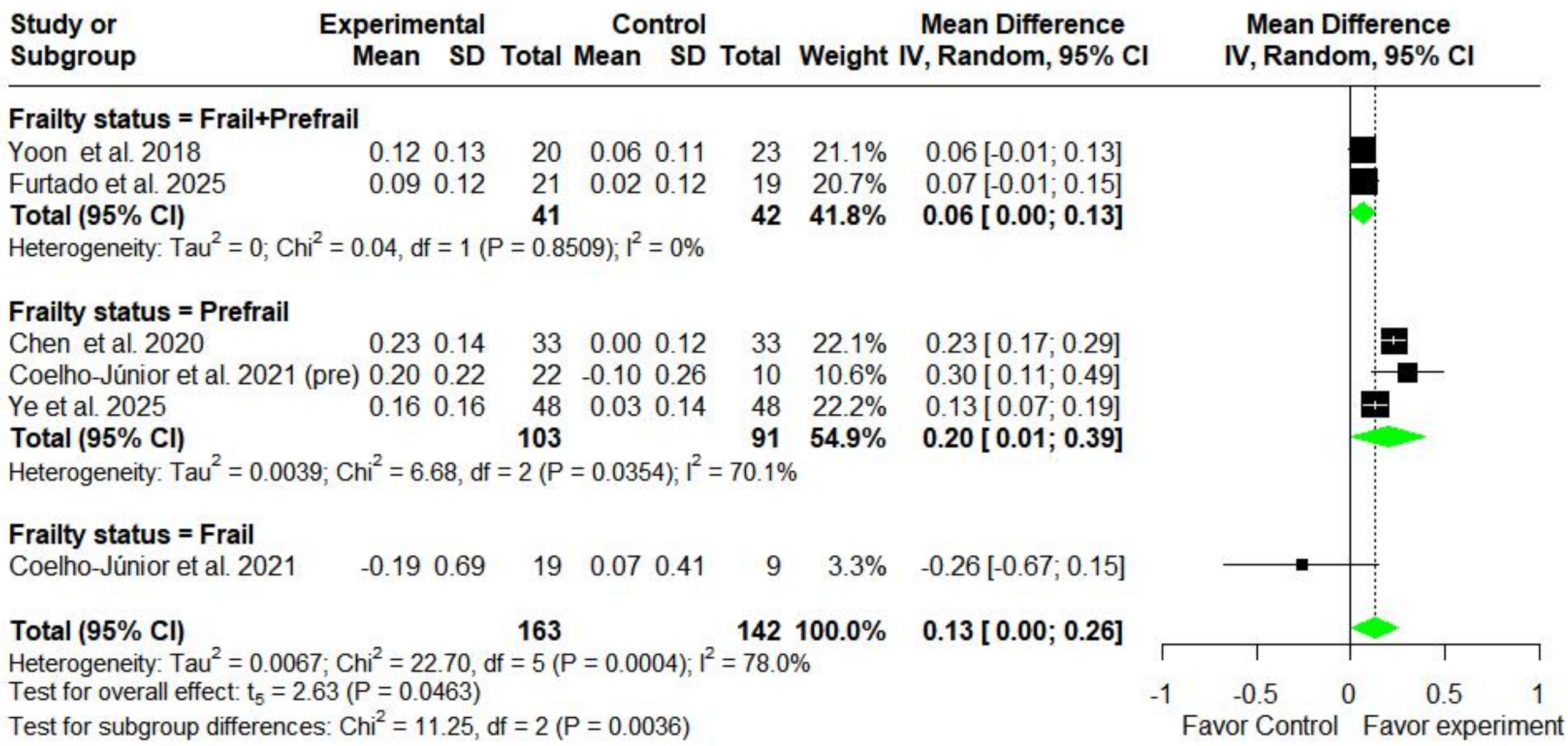

Figure S6.9. Subgroup analysis of gait speed by session duration.

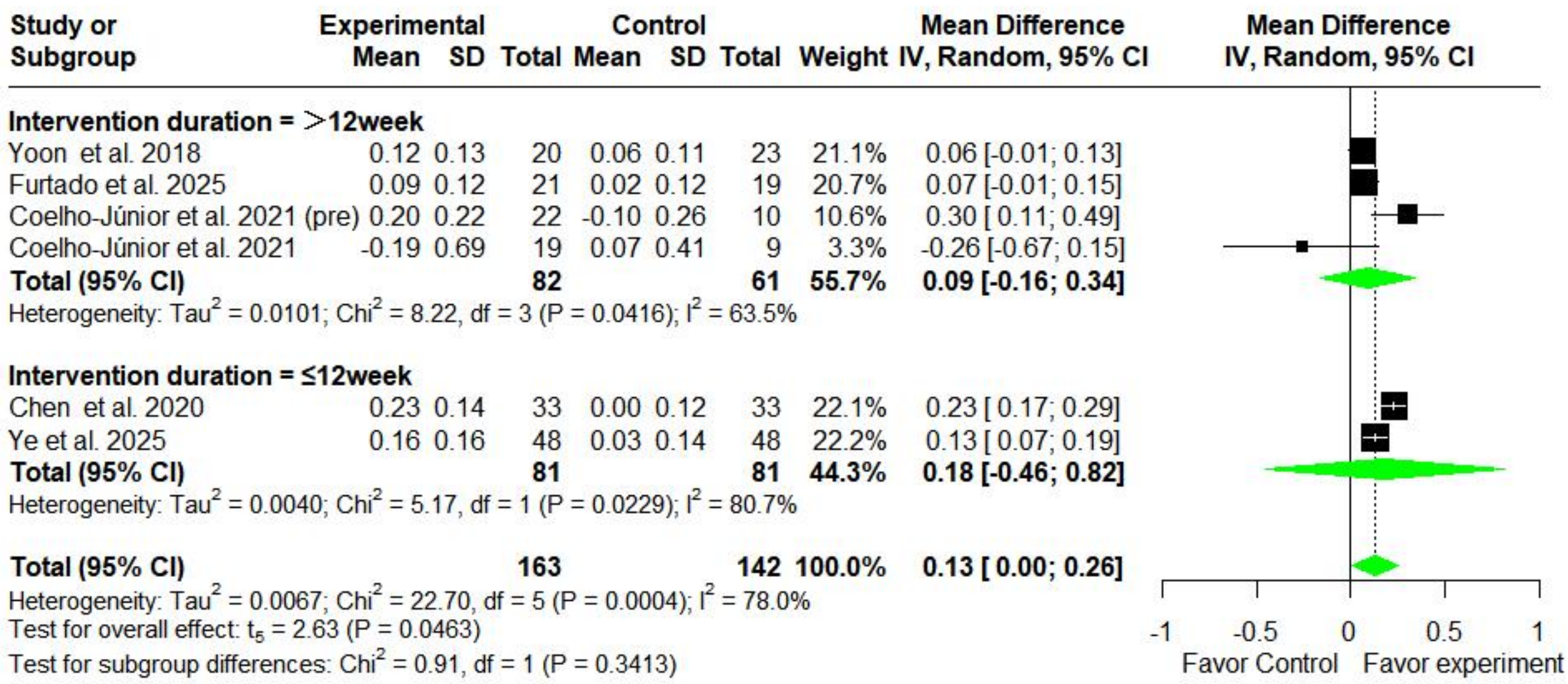

TUG

Figure S6.10. Subgroup analysis of TUG by session duration.

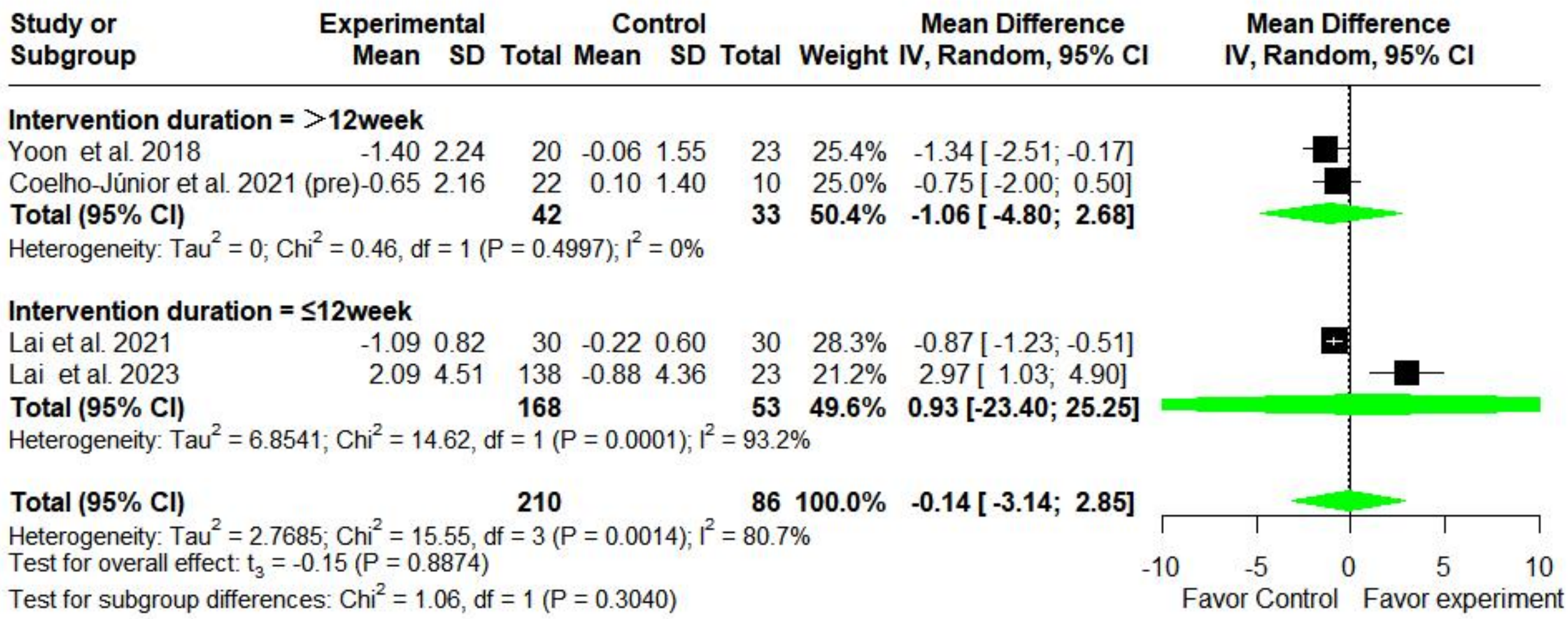

Supplementary Material S7. Sensitivity Analyses

S7.1 Sensitivity Analyses Using Alternative Correlation Assumptions

Figure S7.1. Sensitivity analysis of handgrip strength using assumed correlations of R = 0.3.

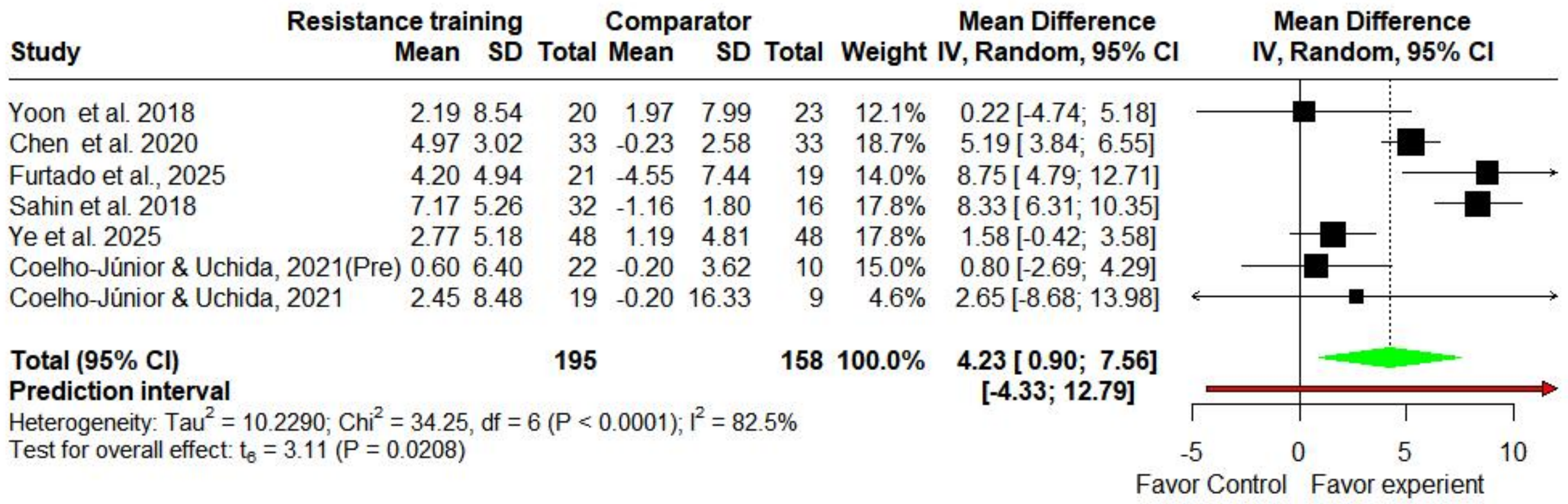

Figure S7.2. Sensitivity analysis of handgrip strength using assumed correlations of R = 0.7.

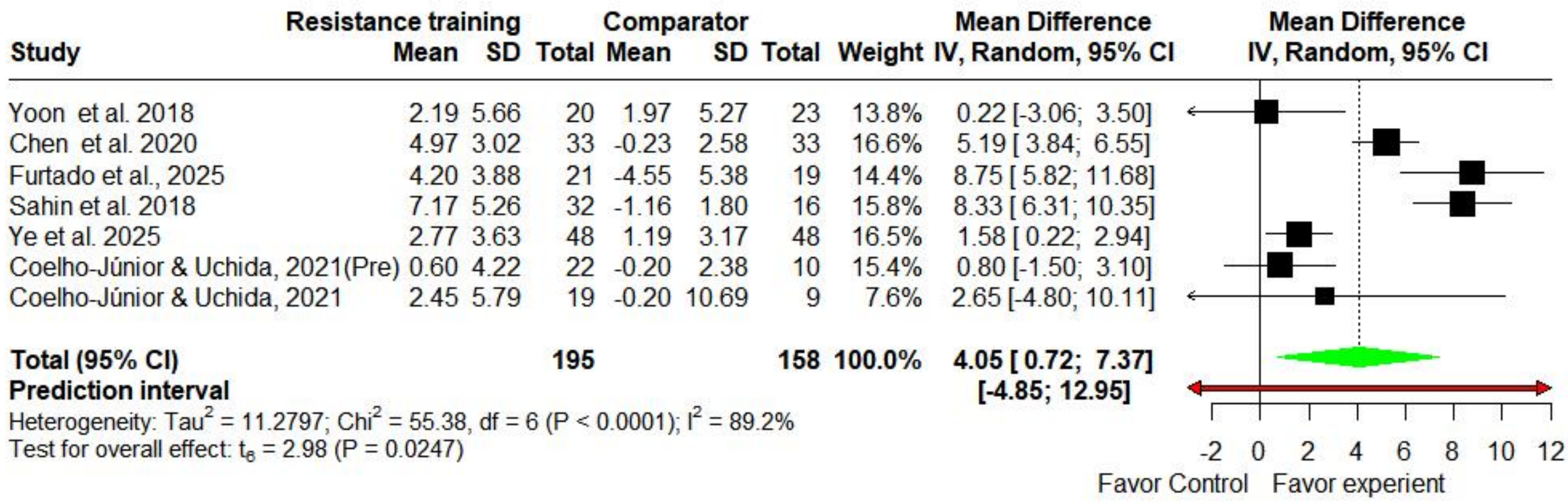

Figure S7.3. Sensitivity analysis of knee extensor strength using assumed correlations of R = 0.3.

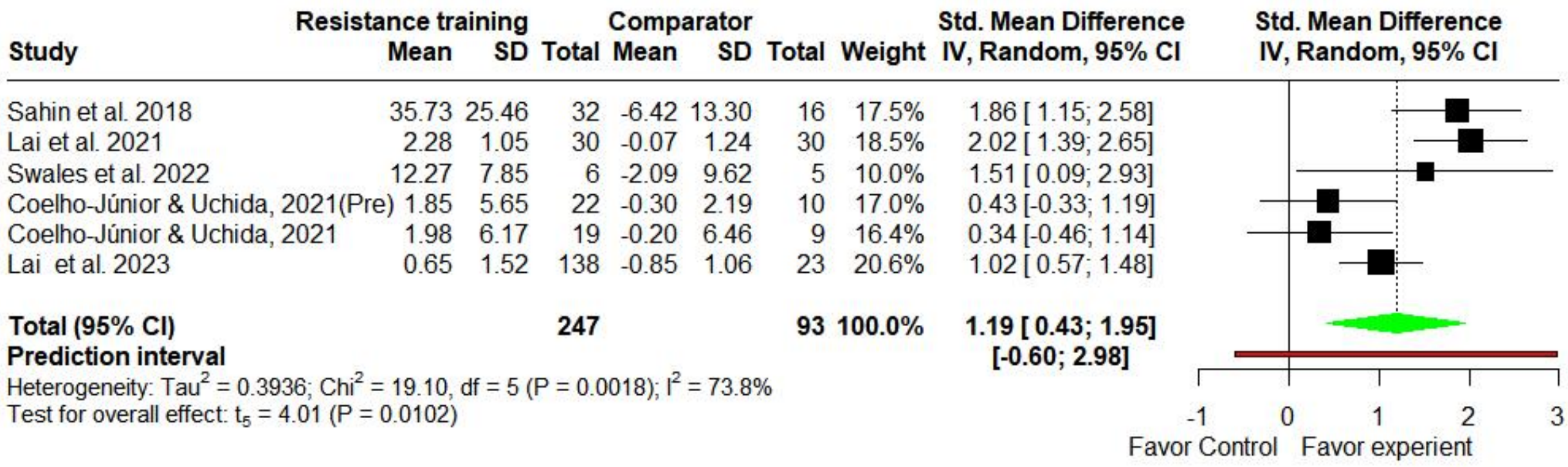

Figure S7.4. Sensitivity analysis of knee extensor strength using assumed correlations of R = 0.7.

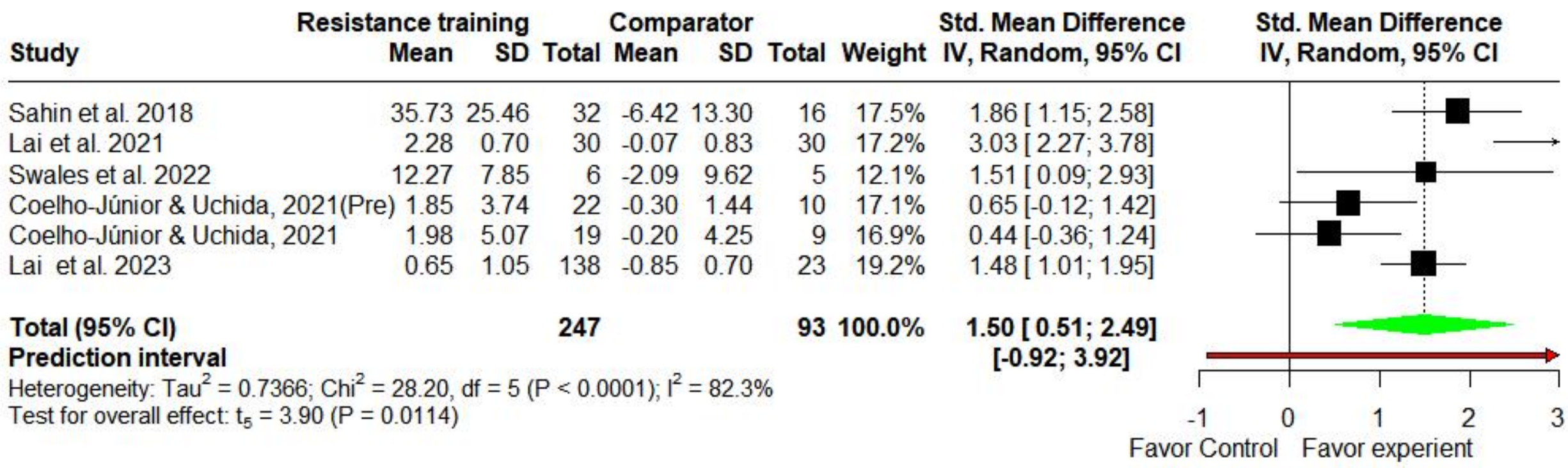

Figure S7.5. Sensitivity analysis of gait speed using assumed correlations of R = 0.3.

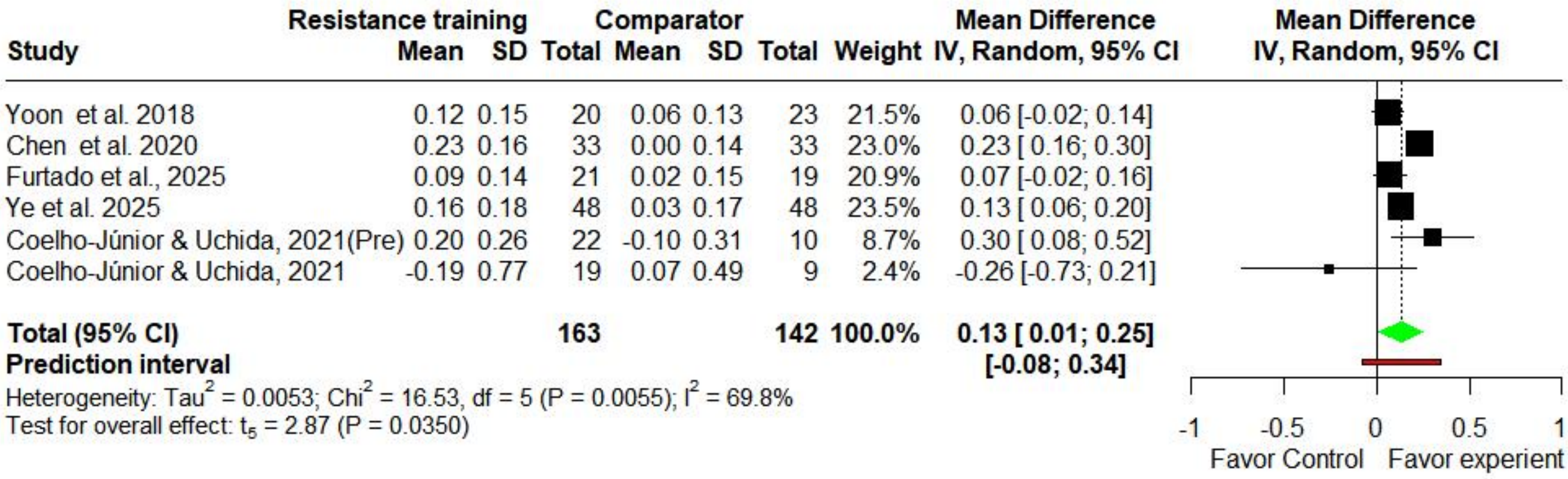

Figure S7.6. Sensitivity analysis of gait speed using assumed correlations of R = 0.7.

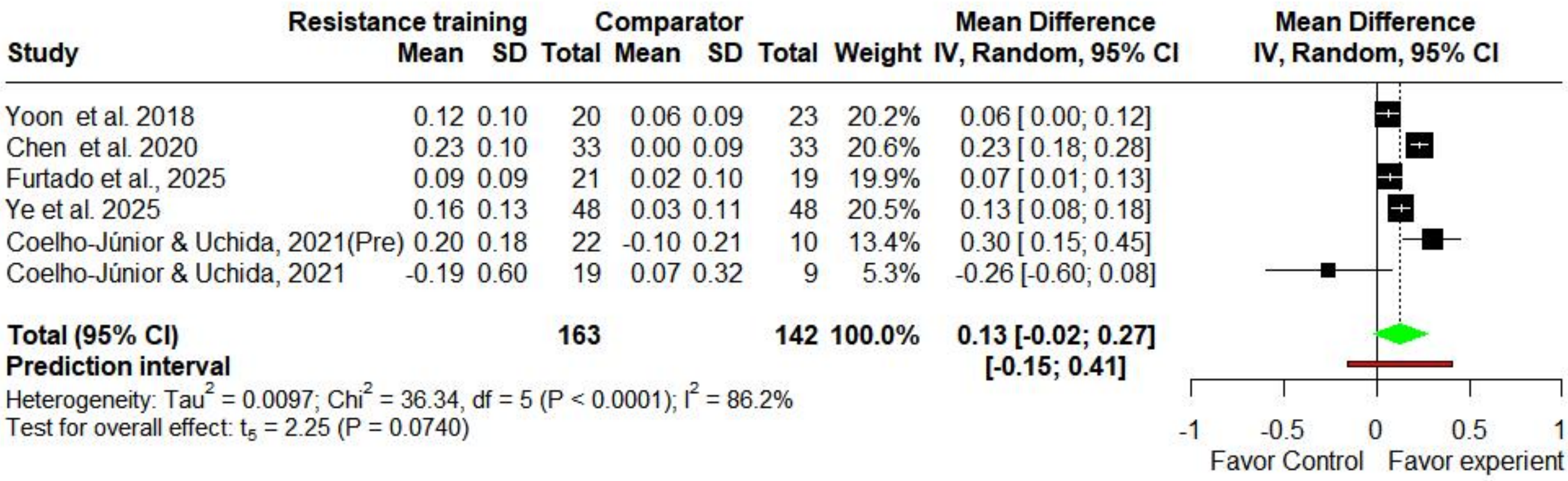

Figure S7.7. Sensitivity analysis of Timed Up and Go using assumed correlations of R = 0.3.

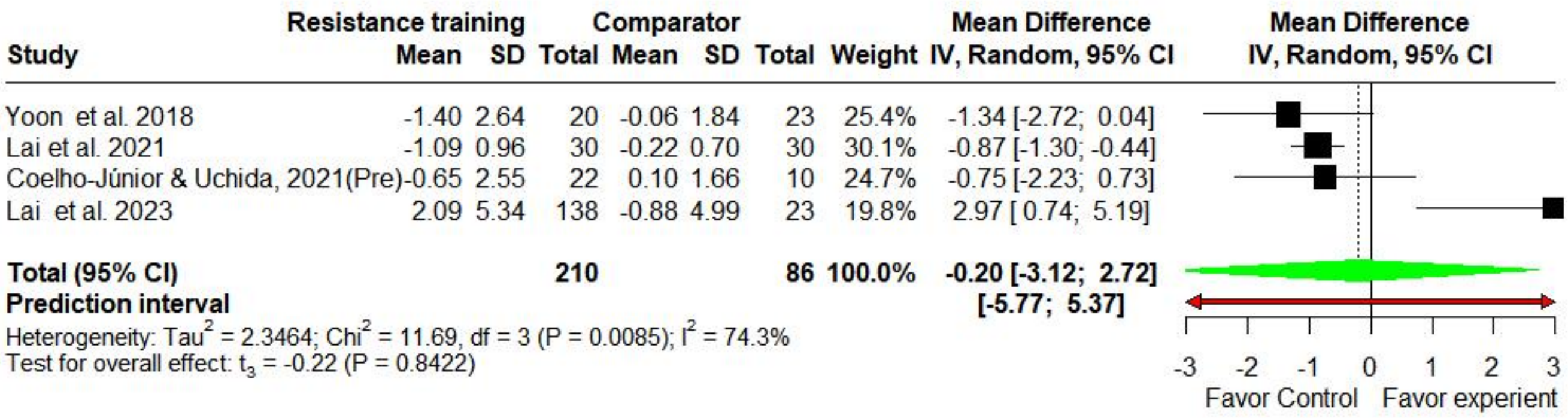

Figure S7.8. Sensitivity analysis of Timed Up and Go using assumed correlations of R = 0.7.

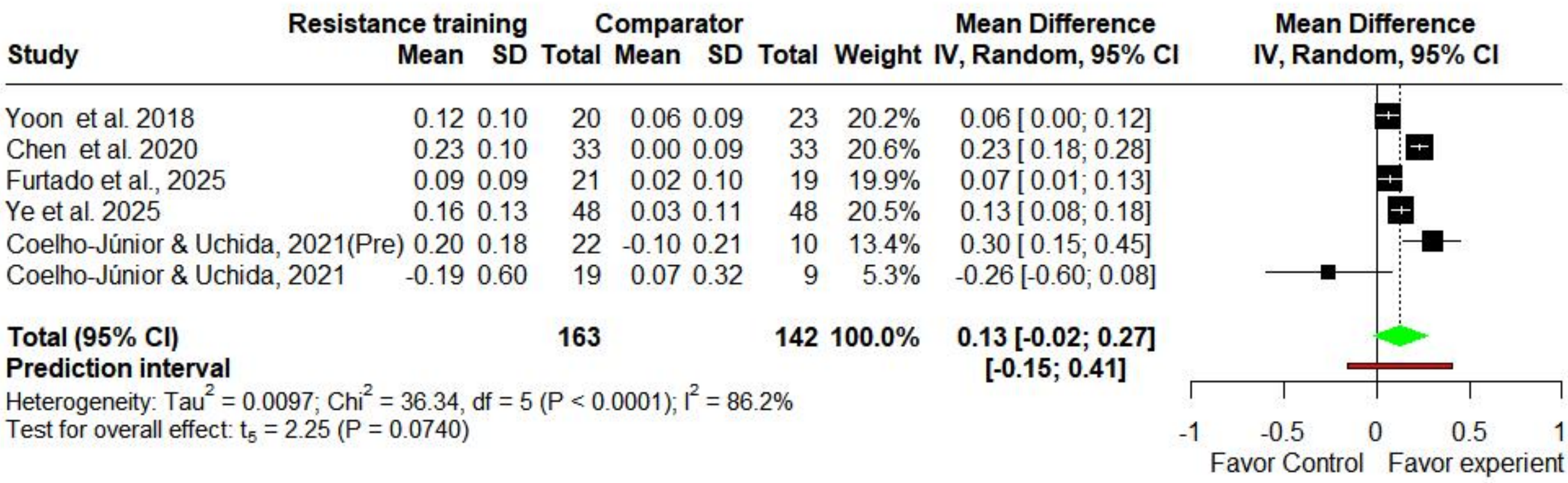

Figure S7.9. Sensitivity analysis of Short Physical Performance Battery using assumed correlations of R = 0.3.

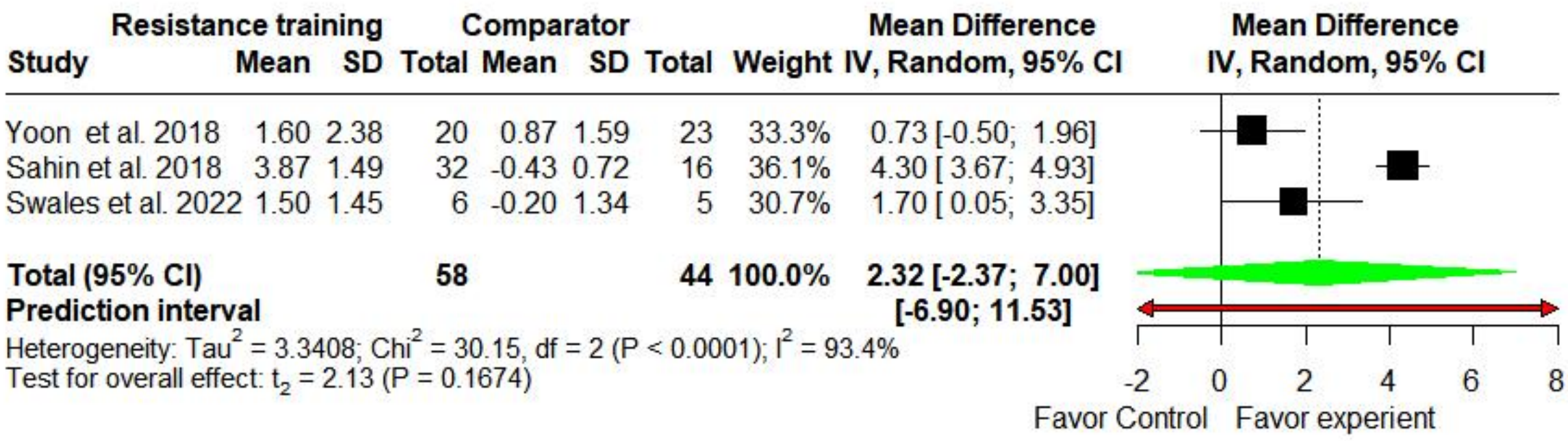

Figure S7.10. Sensitivity analysis of Short Physical Performance Battery using assumed correlations of R = 0.3.

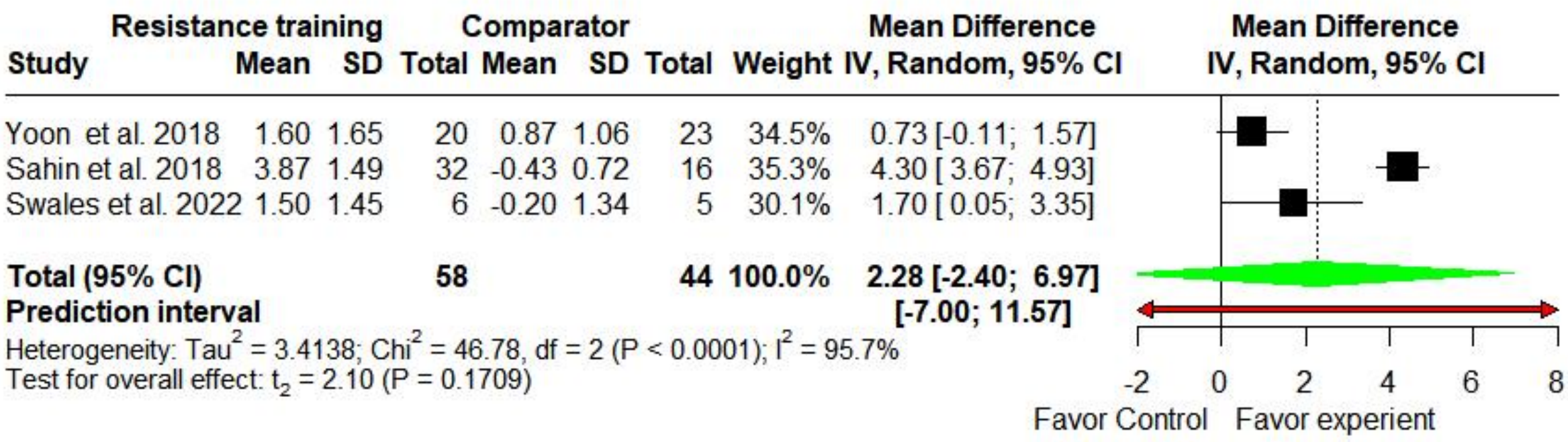

Figure S7.11. Sensitivity analysis of frailty score using assumed correlations of R = 0.3.

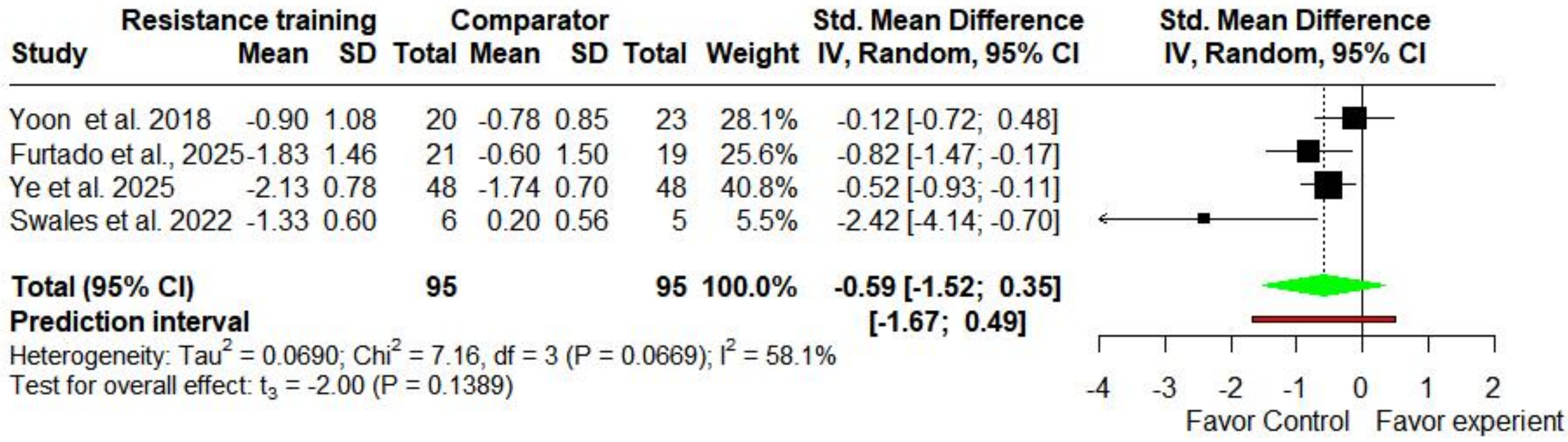

Figure S7.12. Sensitivity analysis of frailty score using assumed correlations of R = 0.7.

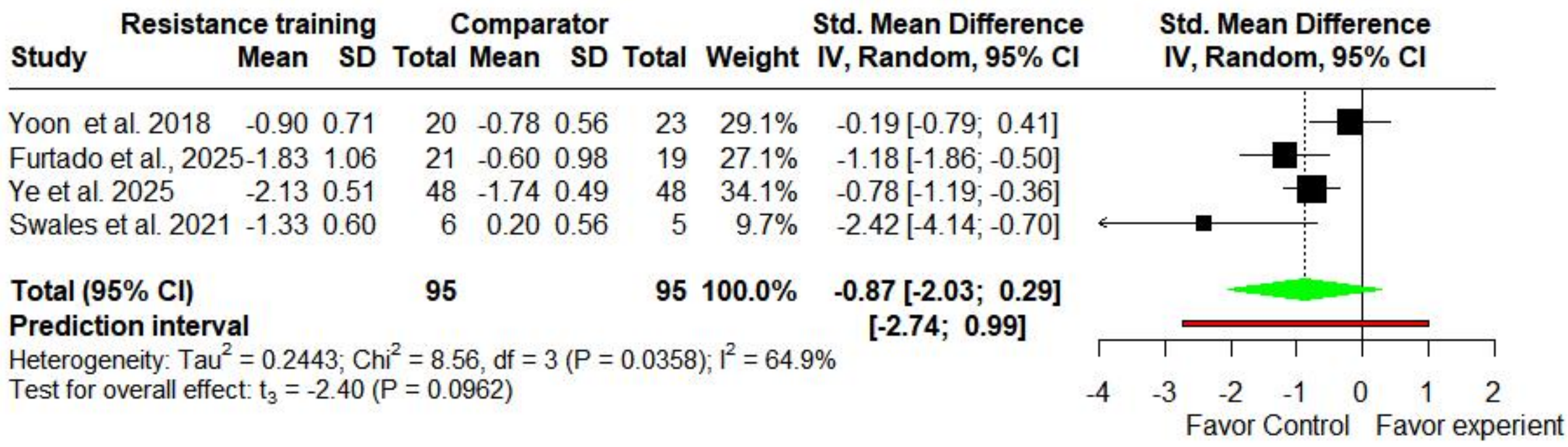

S7.2 Leave-One-Out Sensitivity Analyses

Figure S7.13. Leave-one-out sensitivity analysis for handgrip strength.

Study	P-value	Tau2	Tau	I2	MD	95% CI
Omitting Yoon et al. 2018	0.0210	10.35	3.22	86.7%	4.73	[1.07; 8.39]
Omitting Chen et al. 2020	0.0615	13.59	3.69	87.5%	3.90	[-0.27; 8.07]
Omitting Furtado et al., 2025	0.0511	9.25	3.04	86.0%	3.41	[-0.02; 6.84]
Omitting Coelho-Júnior & Uchida, 2021(Pre)	0.0229	10.50	3.24	85.8%	4.75	[0.98; 8.52]
Omitting Coelho-Júnior & Uchida, 2021	0.0396	11.75	3.43	88.0%	4.23	[0.30; 8.16]
Omitting Sahin et al. 2018	0.0573	8.35	2.89	80.0%	3.31	[-0.15; 6.77]
Omitting Ye et al. 2025	0.0288	11.47	3.39	81.6%	4.67	[0.72; 8.62]
**Total (95% CI)**	**0.0227**	**10.76**	**3.28**	**85.6%**	**4.15**	**[0.81; 7.48]**

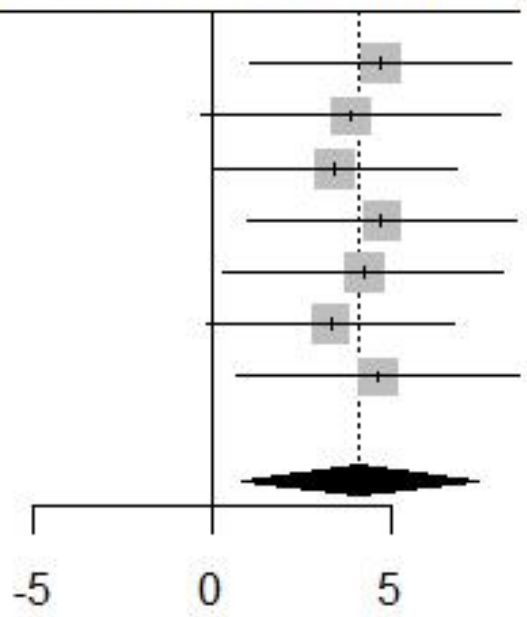

Figure S7.14. Leave-one-out sensitivity analysis for knee extensor strength.

Study	P-value	Tau2	Tau	I2	SMD	95% CI
Omitting Sahin et al. 2018	0.0329	0.55	0.74	79.2%	1.19	[0.16; 2.21]
Omitting Lai et al. 2021	0.0197	0.25	0.50	61.3%	1.07	[0.28; 1.86]
Omitting Swales et al. 2022	0.0284	0.59	0.77	81.7%	1.28	[0.22; 2.33]
Omitting Coelho-Júnior & Uchida, 2021(Pre)	0.0128	0.45	0.67	76.1%	1.47	[0.52; 2.42]
Omitting Coelho-Júnior & Uchida, 2021	0.0101	0.40	0.63	74.7%	1.49	[0.59; 2.39]
Omitting Lai et al. 2023	0.0291	0.66	0.81	81.3%	1.33	[0.22; 2.44]
**Total (95% CI)**	**0.0099**	**0.49**	**0.70**	**77.2%**	**1.30**	**[0.48; 2.13]**

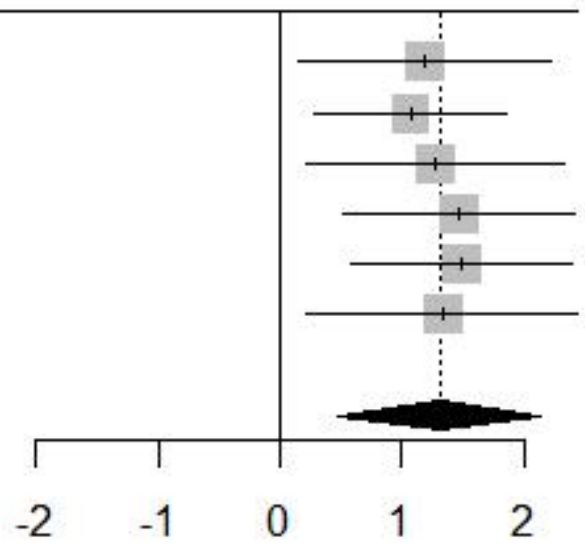

Figure S7.15. Leave-one-out sensitivity analysis for gait speed.

Study	P-value	Tau2	Tau	I2	MD	95% CI
Omitting Yoon et al. 2018	0.0680	< 0.01	0.09	76.9%	0.15	[-0.02; 0.31]
Omitting Chen et al. 2020	0.0675	< 0.01	0.04	60.2%	0.10	[-0.01; 0.21]
Omitting Furtado et al. 2025	0.0810	< 0.01	0.09	79.2%	0.14	[-0.03; 0.32]
Omitting Coelho-Júnior et al. 2021 (pre)	0.0825	< 0.01	0.08	79.7%	0.11	[-0.02; 0.25]
Omitting Coelho-Júnior et al. 2021	0.0271	< 0.01	0.08	79.1%	0.14	[0.03; 0.26]
Omitting Ye et al. 2025	0.1374	0.01	0.11	82.3%	0.13	[-0.06; 0.32]
**Total (95% CI)**	**0.0463**	**< 0.01**	**0.08**	**78.0%**	**0.13**	**[0.00; 0.26]**

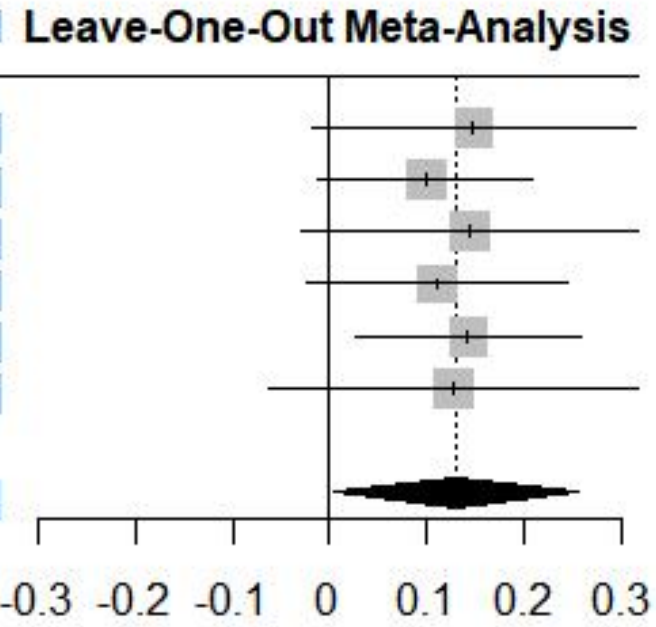

Figure S7.16. Leave-one-out sensitivity analysis for Timed Up and Go.

Study	P-value	Tau2	Tau	I2	MD	95% CI
Omitting Yoon et al. 2018	0.8261	3.77	1.94	86.3%	0.30	[-4.94; 5.55]
Omitting Coelho-Júnior et al. 2021 (pre)	0.9344	4.55	2.13	87.1%	0.12	[-5.57; 5.82]
Omitting Lai et al. 2021	0.8984	4.48	2.12	86.1%	0.19	[-5.50; 5.88]
Omitting Lai et al. 2023	0.0110	0	0	0%	-0.90	[-1.31; -0.49]
**Total (95% CI)**	**0.8874**	**2.77**	**1.66**	**80.7%**	**-0.14**	**[-3.14; 2.85]**

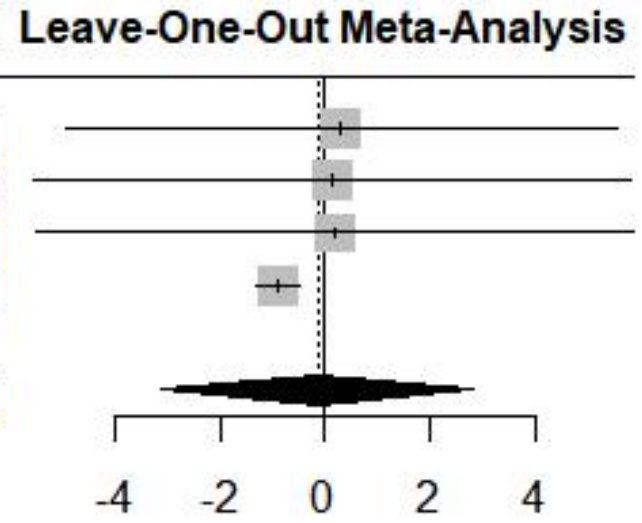

Figure S7.17. Leave-one-out sensitivity analysis for Short Physical Performance Battery.

Study	P-value	Tau2	Tau	I2	MD	95% CI
Omitting Yoon et al. 2018	0.2507	2.98	1.72	88.0%	3.12	[-13.34; 19.57]
Omitting Sahin et al. 2018	0.2613	0	0	0%	1.01	[-4.58; 6.61]
Omitting Swales et al. 2022	0.3898	6.18	2.49	96.9%	2.54	[-20.14; 25.22]
**Total (95% CI)**	**0.1692**	**3.38**	**1.84**	**94.5%**	**2.30**	**[-2.39; 6.99]**

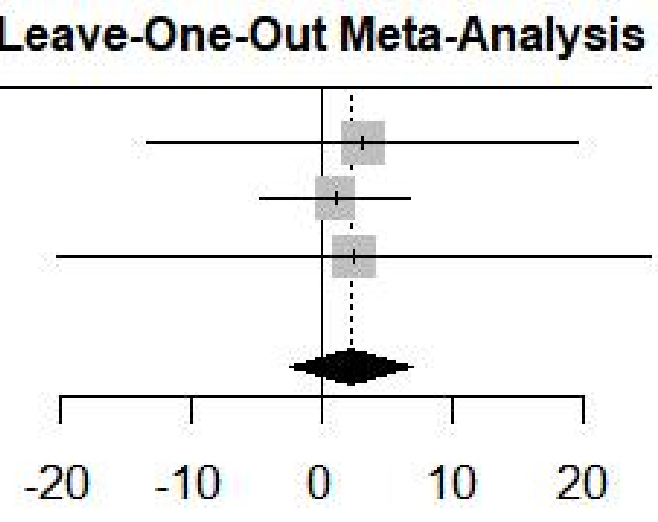

Figure S7.18. Leave-one-out sensitivity analysis for frailty score.

Study	P-value	Tau2	Tau	I2	MD	95% CI
Omitting Yoon et al. 2018	0.1096	0.33	0.57	83.7%	-0.98	[-2.51; 0.55]
Omitting Furtado et al., 2025	0.2652	0.43	0.66	82.7%	-0.64	[-2.44; 1.16]
Omitting Ye et al. 2025	0.1689	0.49	0.70	84.2%	-0.93	[-2.81; 0.96]
Omitting Swales et al. 2022	0.2391	0.15	0.39	63.7%	-0.49	[-1.78; 0.79]
**Total (95% CI)**	**0.1061**	**0.35**	**0.59**	**80.1%**	**-0.76**	**[-1.82; 0.30]**

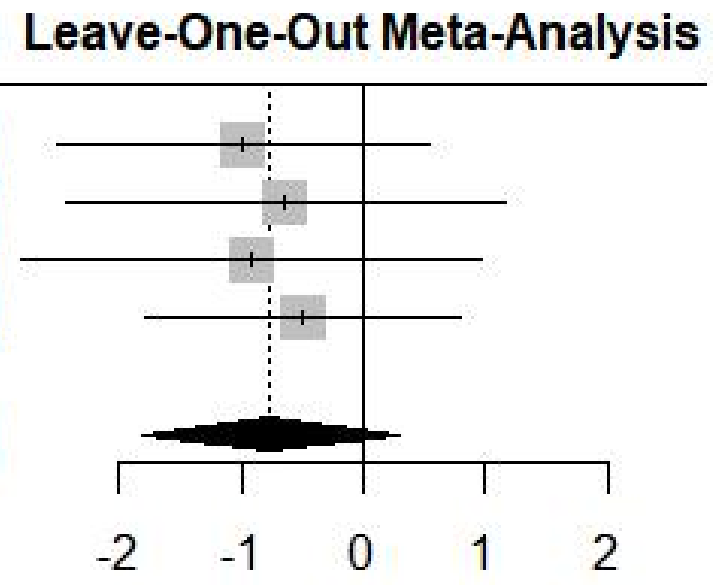

S7.3 Sensitivity Analyses Excluding Studies With Active Control Conditions

S7.3.1 Handgrip Strength

Figure S7.19. Sensitivity analysis for handgrip strength excluding studies with active control conditions.

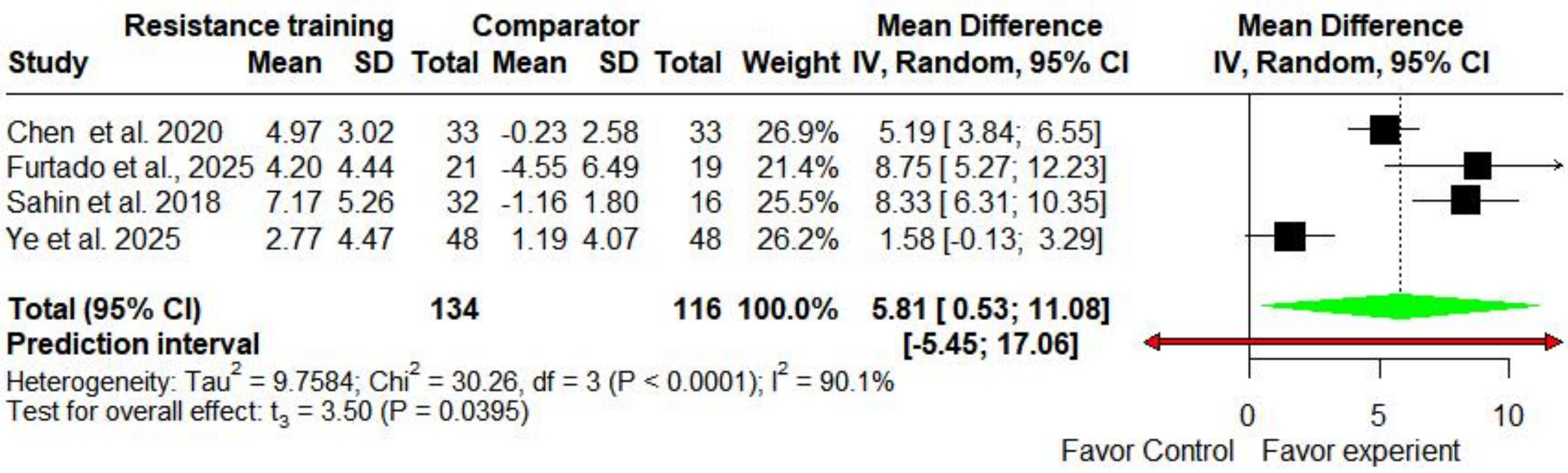

Figure S7.20. Sensitivity analysis for gait speed excluding studies with active control conditions.

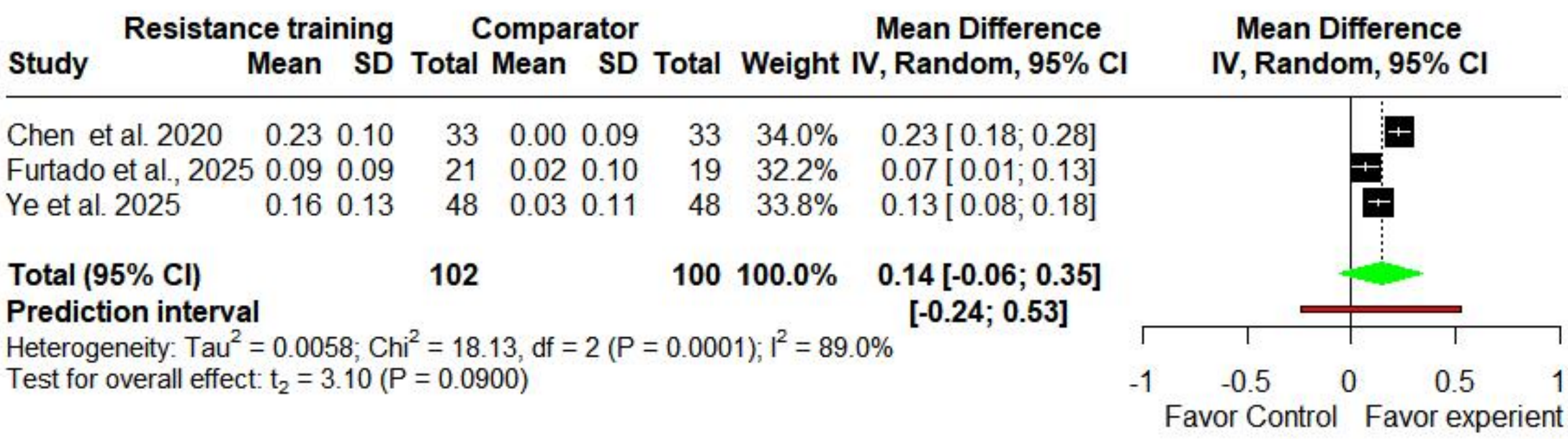

Supplementary Material S8. Funnel plots

Figure S8.1. Funnel plot for knee extensor strength (KES).

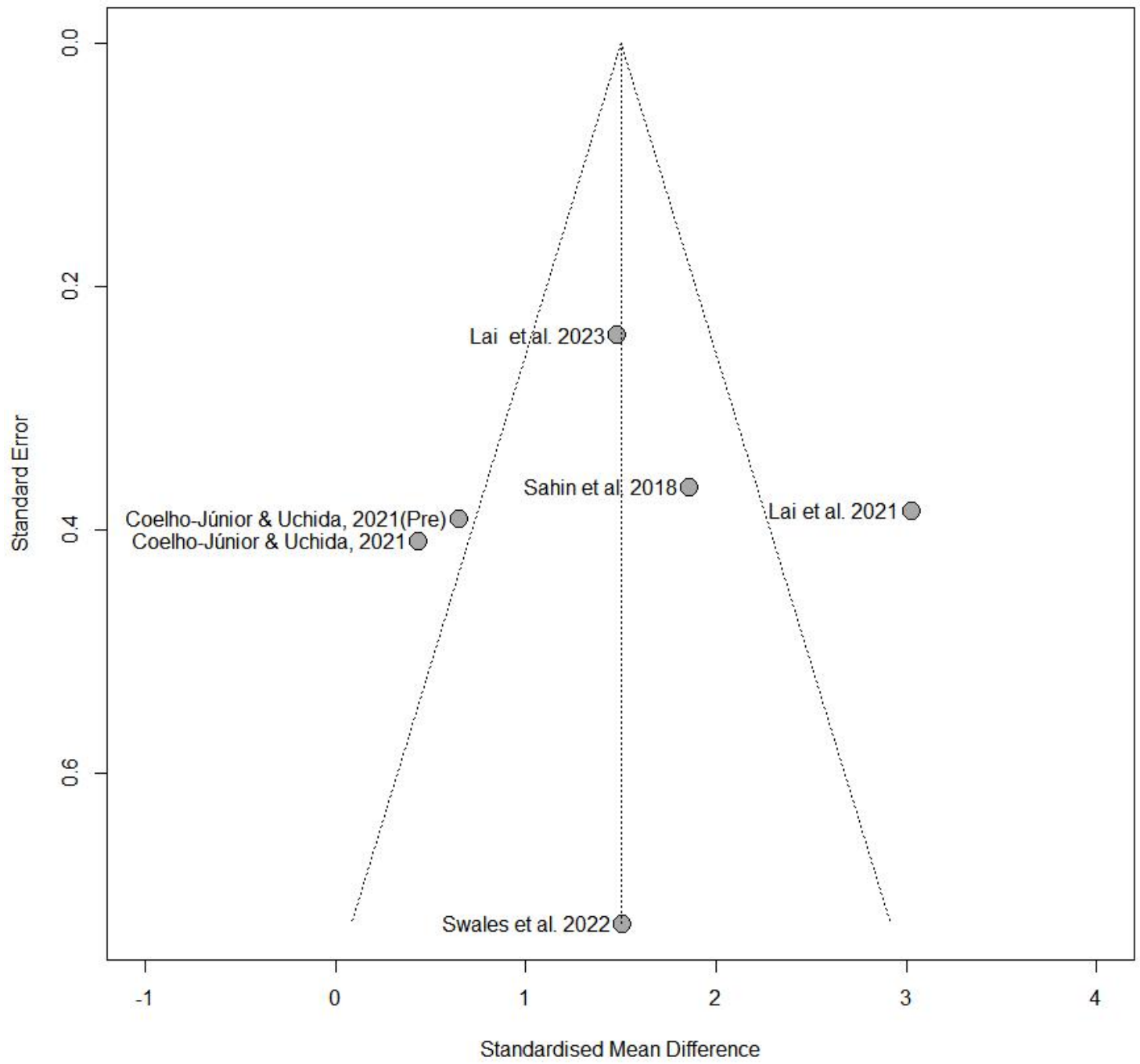

Figure S8.2. Funnel plot for handgrip strength.

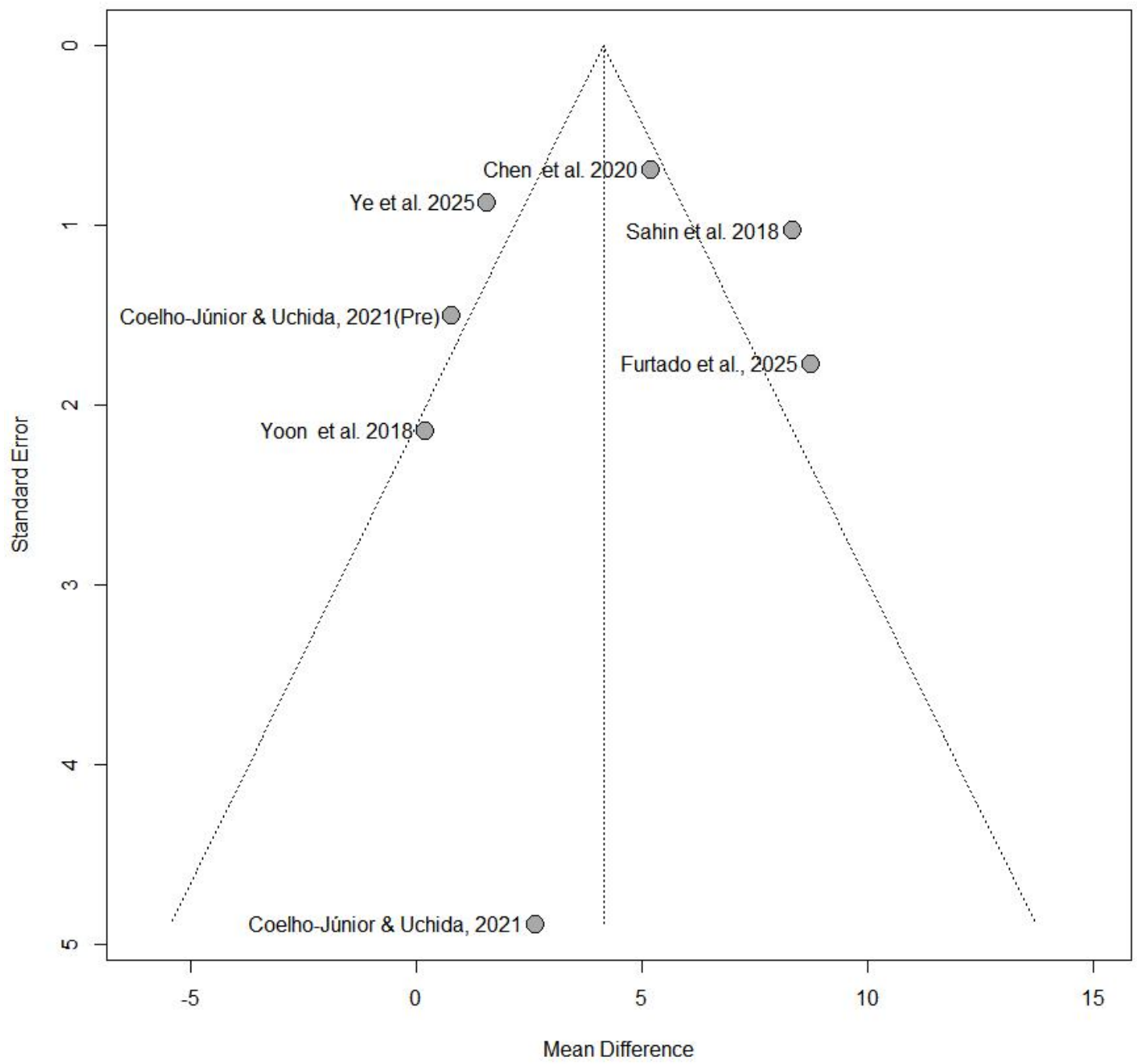

Figure S8.3. Funnel plot for Short Physical Performance Battery (SPPB).

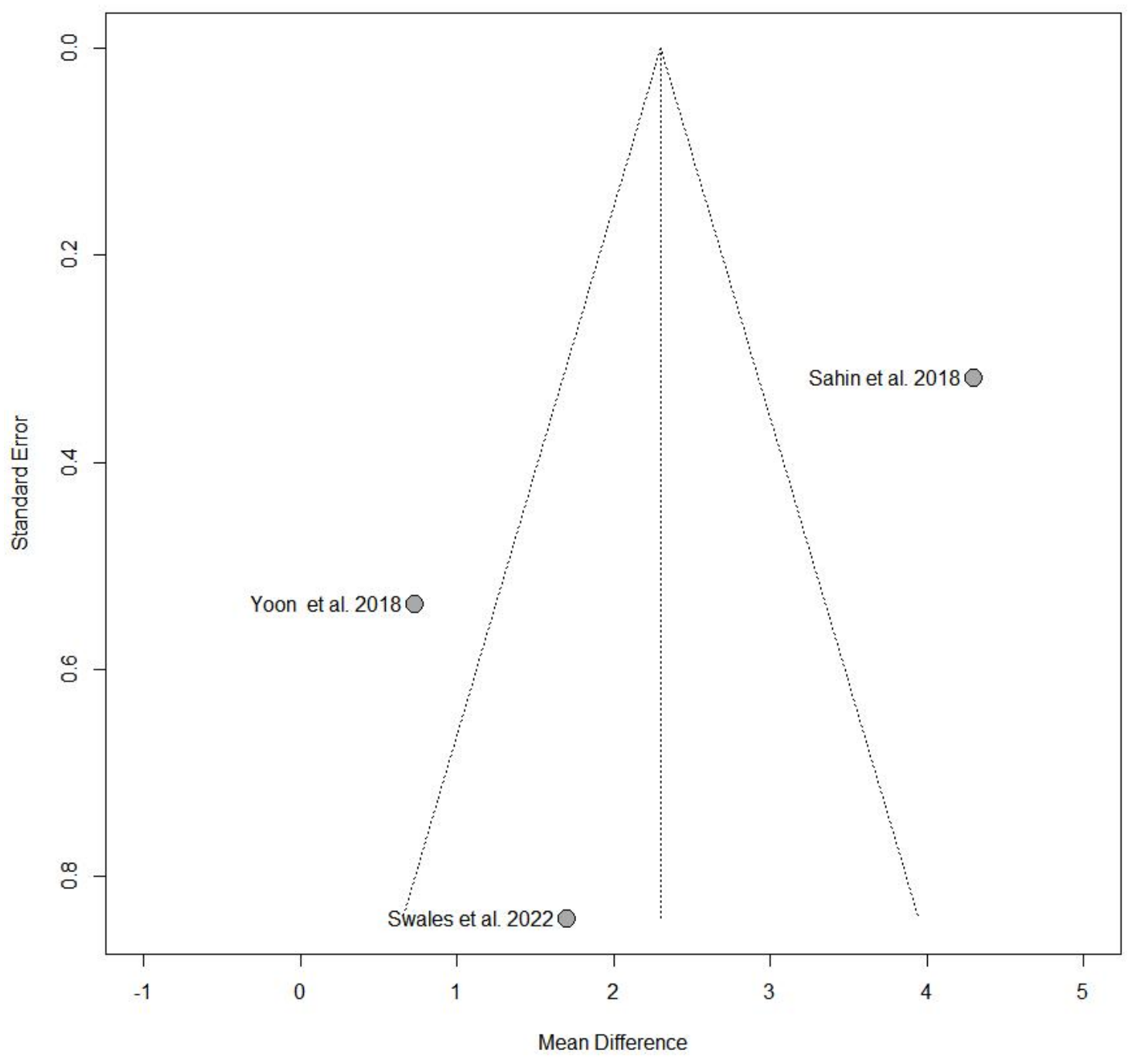

Figure S8.4. Funnel plot for Timed Up and Go (TUG).

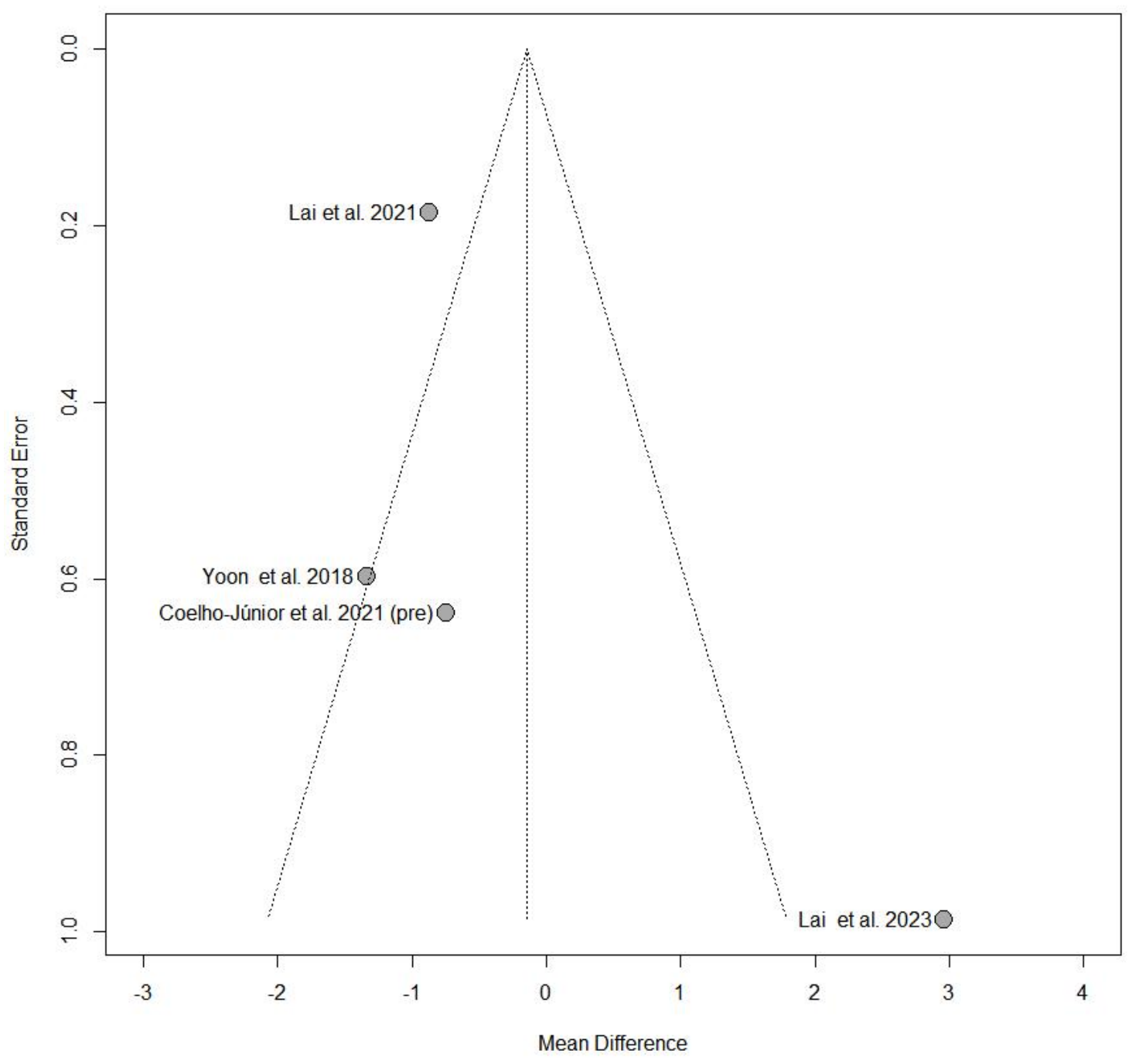

Figure S8.5. Funnel plot for gait speed.

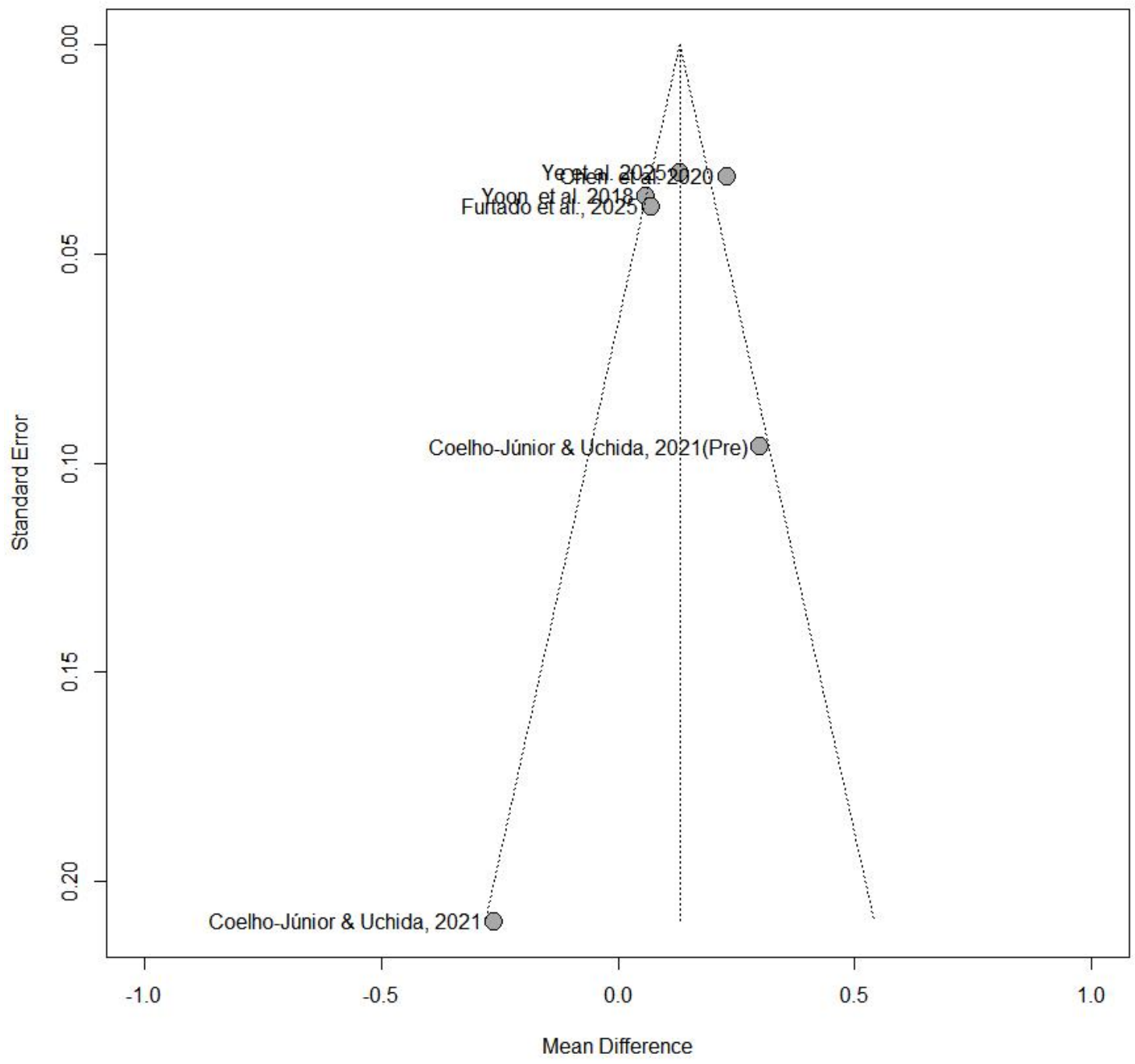

Figure S8.6. Funnel plot for frailty score.

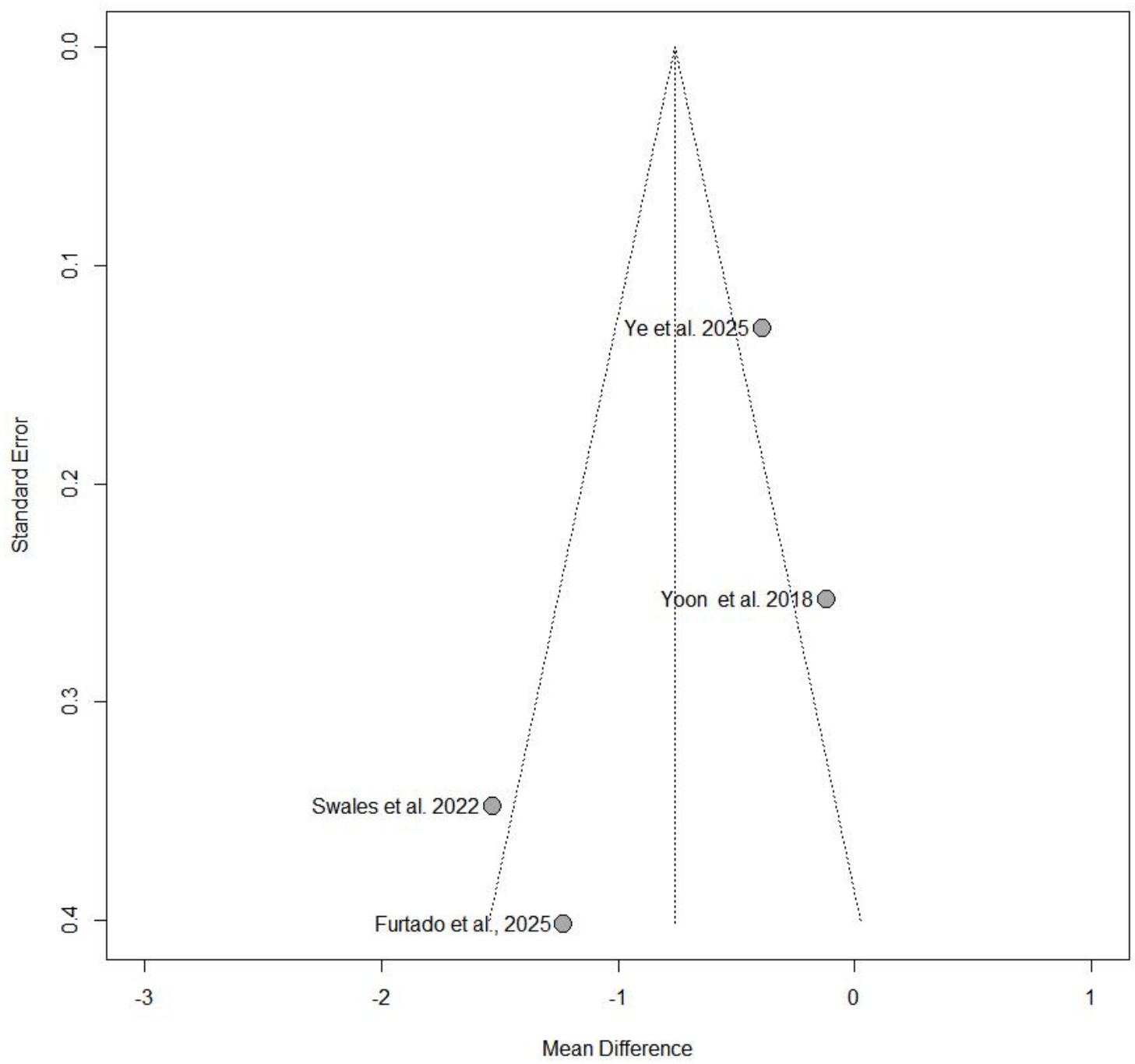

Supplement: Supplementary file 1 [file Data_Sheet_1.pdf]
